# Setting expected timelines of fished population recovery for the adaptive management of a marine protected area network

**DOI:** 10.1002/eap.1949

**Published:** 2019-07-26

**Authors:** Katherine A. Kaplan, Lauren Yamane, Louis W. Botsford, Marissa L. Baskett, Alan Hastings, Sara Worden, J. Wilson White

**Affiliations:** ^1^ Department of Evolution and Ecology, Coastal and Marine Sciences Institute University of California Davis One Shields Avenue Davis California 95616 USA; ^2^ California Department of Fish and Wildlife Marine Region 350 Harbor Boulevard Belmont California 94002 USA; ^3^ Department of Wildlife Fish and Conservation Biology University of California Davis One Shields Avenue Davis California 95616 USA; ^4^ Department of Environmental Science and Policy University of California Davis One Shields Avenue Davis California 95616 USA; ^5^ Department of Fisheries and Wildlife Coastal Oregon Marine Experiment Station Oregon State University Newport Oregon 97365 USA

**Keywords:** adaptive management, age structure, fisheries, linear population model, marine reserves, transient dynamics

## Abstract

Adaptive management of marine protected areas (MPAs) requires developing methods to evaluate whether monitoring data indicate that they are performing as expected. Modeling the expected responses of targeted species to an MPA network, with a clear timeline for those expectations, can aid in the development of a monitoring program that efficiently evaluates expectations over appropriate time frames. Here, we describe the expected trajectories in abundance and biomass following MPA implementation for populations of 19 nearshore fishery species in California. To capture the process of filling in the age structure truncated by fishing, we used age‐structured population models with stochastic larval recruitment to predict responses to MPA implementation. We implemented both demographically open (high larval immigration) and closed (high self‐recruitment) populations to model the range of possible trajectories as they depend on recruitment dynamics. From these simulations, we quantified the time scales over which anticipated increases in abundance and biomass inside MPAs would become statistically detectable. Predicted population biomass responses range from little change, for species with low fishing rates, to increasing by a factor of nearly seven, for species with high fishing rates before MPA establishment. Increases in biomass following MPA implementation are usually greater in both magnitude and statistical detectability than increases in abundance. For most species, increases in abundance would not begin to become detectable for at least 10 years after implementation. Overall, these results inform potential indicator metrics (biomass), potential indicator species (those with a high fishing : natural mortality ratio), and time frame (>10 yr) for MPA monitoring assessment as part of the adaptive management process.

## Introduction

Marine protected areas (MPAs) are locations in which fishing and other extractive activities are limited or prohibited, often with multiple objectives ranging from preservation of fishery resources to promoting ecosystem integrity (Leslie 2005, Edgar et al. 2007, Klein et al. 2008, Halpern et al. 2010). Frequently, MPAs are implemented with a goal of employing an adaptive management process (Walters and Holling 1990), in which the expected response to a management action is determined, the system is monitored to see whether those expectations are met, and management is updated accordingly (Atkinson et al. 2004, McCarthy and Possingham 2007, Lyons et al. 2008, Lindenmayer and Likens 2009, 2010). However, a key piece that is often missing in this process is the quantitative prediction of the expected population response to protection (White et al. 2011). Recent advances in our understanding of population responses to MPAs can inform this process. Specifically, we now have both empirical assessments of population increases within MPAs (Lester et al. 2009, Babcock et al. 2010, Claudet et al. 2010, Edgar et al. 2014) and an improved theoretical understanding of short‐term population dynamics following MPA implementation (Moffitt et al. 2011, White et al. 2013a, Hopf et al. 2015).

Generally, one expects populations within MPAs to increase in abundance following MPA implementation. However, population responses can vary widely depending on a variety of factors including the time frame of assessment, environmental stochasticity, species life history, prior levels of fishing pressure, species interactions, habitat loss, and enforcement failures (Halpern and Warner 2002, Jones et al. 2004, Micheli et al. 2004, Babcock et al. 2010, White et al. 2011, 2013a). Disentangling the contributions of these drivers of population responses to MPAs is necessary for adaptive management to determine whether knowledge updates or management changes are necessary. One approach to disentangling such contributions is to compare monitoring data to expectations from population models that account for different drivers of population responses to MPAs.

Positive population responses to MPAs arise from the cessation of fishing mortality accompanying the implementation of an MPA (provided the MPA is large enough relative to the spatial scale of fish movement). Eliminated fishing mortality initially produces a “filling in” effect in a population's age and size distribution as fish live longer, grow larger, and the population returns to the unfished age (or size) distribution (White et al. 2013a, Baskett and Barnett 2015). The time frame and magnitude of this response inevitably depend on a population's fishing history, life history, and dispersal dynamics. If the population in the MPA is largely demographically “open” (i.e., most larvae settling in the MPA were spawned elsewhere), the long‐term increase in population abundance should depend on both the pre‐MPA fishing rate, *F*, and the population's natural mortality rate, *M*. Specifically, the eventual population abundance, relative to the pre‐MPA state, should be (*M* + *F*)/*M*, and it should approach that abundance at the rate *e* ^–*M*^ (White et al. 2013a). Alternatively, if a substantial fraction of the offspring produced in an MPA returns to the same MPA (or MPA network) after the pelagic larval phase (i.e., the population is demographically “closed”), the increases in abundance of older, more fecund fish will eventually lead to an increase in larval recruitment in the MPA. Time lags in such “closed” populations can lead to short‐term (transient) oscillations in abundance before eventually increasing or decreasing. In general, closed populations that have lower natural mortality rates, older ages at maturity, or have been fished more intensely prior to MPA implementation are more likely to exhibit higher‐amplitude oscillations during the transient (White et al. 2013a). Either type of dynamic, open or closed, could stymie MPA managers attempting to evaluate species responses to MPAs. In the “open” scenario, expected population increases may be small (e.g., for a lightly fished population). In the “closed” scenario, populations that will eventually increase may initially exhibit short‐term declines (White et al. 2013a).

Environmental stochasticity further complicates setting realistic expectations for detectable population responses to MPAs over short time scales (White et al. 2011). A common manifestation of environmental stochasticity in marine systems is interannual variability in larval recruitment, driven by variation in ocean conditions. The resulting year‐to‐year pulses and droughts in recruitment create uncertainty in how increases in abundance and biomass feed back into population dynamics (Fogarty et al. 1991, Planque et al. 2010). Therefore, efforts to set realistic expectations for changes in population biomass and abundance must account for the ability to detect population changes inside the MPA in the presence of environmental stochasticity.

Current evaluations and projections for MPA responses typically focus on post‐hoc analysis of observed trajectories (Pandolfi et al. 2003, McClanahan and Graham 2005, McClanahan et al. 2007, Molloy et al. 2009, Babcock et al. 2010). Meta‐analyses of such studies have been used to set broad expectations for population recoveries based on typical factors that influence MPA responses (Christie et al. 2009, Lester et al. 2009, Fox et al. 2012, Edgar et al. 2014, Gill et al. 2017). These meta‐analyses demonstrate that there is great variation in population responses to MPAs, both among different species in the same MPA and for the same species in different MPAs (Christie et al. 2009, Lester et al. 2009, Fox et al. 2012, Edgar et al. 2014, Gill et al. 2017). Therefore, the unique context of specific MPAs and their associated species is central to tailoring realistic expectations for their implementation. Creating such tailored expectations moves MPA analysis beyond post‐hoc evaluation by providing more context‐dependent (i.e., species and location), ante hoc expectations for the adaptive management process described above. Existing MPA theory (White et al. 2013b; reviewed by Gerber et al. 2003, Gaines et al. 2010) typically provides quantitative expectations of long‐term (equilibrium) outcomes. Extending this theory to model short‐term responses allows better comparison to monitoring data on management‐relevant time scales. Model outputs can then help identify indicator metrics, species, and time frames as well as allow evaluation of whether MPAs are working as expected in an adaptive management framework.

California's MPA network, consisting of 124 individual coastal MPAs established or expanded between 2003 and 2013 (Kirlin et al. 2013, Botsford et al. 2014), provides an ideal study system. In particular, the wide variation in life history traits of species protected in the network enables evaluation of a diversity of species‐specific projected responses. The legislation that led to the creation of these MPAs, with ultimate goals ranging from sustaining economically important populations to promoting biodiversity conservation, mandates that this network be managed adaptively (Botsford et al. 2014).

Two post hoc studies focused on the Channel Islands MPAs (10 MPAs surrounding four islands off the coast of southern California; implemented in 2003) have documented high variability among species in post‐2003 population trajectories. Overall, species harvested by fisheries have increased in abundance inside MPAs, while non‐targeted species have not (Hamilton et al. 2010, Caselle et al. 2015). However, individual harvested species, which vary substantially in life history and fishing history, ranged from exhibiting large increases to none at all (Hamilton et al. 2010, Caselle et al. 2015). Additionally, a study assessing central California MPAs (implemented in 2007) found that fish size and abundance did not increase within a new MPA (relative to a reference site) within the first 7 yr, but that those metrics were greater in an older, previously existing MPA (Starr et al. 2015). These studies reinforce the need to understand expectations for the time scale and magnitude of population increase after MPAs are implemented, and how those vary among species.

Here, we develop age‐structured population models to create a timeline of expected trajectories for 19 harvested nearshore fish and invertebrate populations in California MPAs based on life history characteristics and estimates of pre‐MPA harvest rates. We use these models to quantify how population abundance and biomass compare in the magnitude and time scale of their expected responses to MPA implementation under both open and closed population dynamics. We focus on the length of time during which transient dynamics have the potential to dominate population responses, which informs management questions such as how long to monitor before expecting to see detectable MPA effects and how much change in abundance or biomass we might expect from different species. To provide insight into systems beyond California we use regressions to explore how differences in life‐history, prior fishing, and stochastic recruitment affect the time course of detectability of responses to protection in an MPA. The over‐arching framework and analytic approach we present could be applied, with relevant tailoring to local dynamics, in any location to guide the adaptive management of MPAs.

## Methods

We used age‐structured population models to characterize the expected timeline of population dynamics after fishing ceases inside an MPA. These models portray how abundance increases from the “filling in” of the age structure that has been truncated by fishing. Prior work has shown that these dynamics depend on the degree of demographic connectivity between the focal MPA and other MPAs (or fished habitats) and on the amount of variability in larval recruitment (White and Rogers‐Bennett 2010, White et al. 2013a). To represent the two extreme bounds of demographic connectivity, we explore the post‐MPA dynamics of both an open population and a closed population. In order to explore the role of variable larval recruitment, we analyze both deterministic and stochastic versions of the open population model. In our analysis of open populations, we quantify the magnitude, rate, and variability of the possible increase in both abundance and biomass inside MPAs. In our analyses of the closed populations, we quantify the magnitude and duration of potential short‐term, transient oscillations that can obscure the eventual long‐term trajectory of the population.

### Study species

We assessed 19 species targeted by nearshore fisheries with a wide range of life histories (Table 2). Of those 19 species, 12 are species of rockfish, genus *Sebastes* (Scorpaenidae): kelp rockfish (*S. atrovirens*), blue rockfish (*S. mystinus*), black rockfish (*S. melanops*), gopher rockfish (*S. carnatus*), brown rockfish (*S. auriculatus*), copper rockfish (*S. caurinus*), yellowtail rockfish (*S. flavidus*), vermilion rockfish (*S. miniatus*), bocaccio (*S. paucispinis*), China rockfish (*S. nebulosus*), black and yellow rockfish (*S. chrysomelas*), and olive rockfish *(S. serranoides*). Two are species of Hexagrammidae: lingcod (*Ophiodon elongates*) and kelp greenling (*Hexagrammos decagrammus*). Other species are California scorpionfish (*Scorpaena guttata*; Scorpaenidae); cabezon (*Scorpaenichthys marmoratus*; Cottidae); California sheephead (*Semicossyphus pulcher*; Labridae); kelp bass (*Paralabrax clathratus*; Serranidae); and an invertebrate, red sea urchin (*Mesocentrotus franciscanus*). These species reside in rocky reef and kelp forest habitats along the California coast, though some are found only in warmer southern waters (kelp bass, California sheephead). All of these species have been heavily fished since the 1970s‐1980s and were subjects of concern during MPA planning in California, though harvest on some has been restricted in recent years, allowing stocks to rebuild (e.g., bocaccio; see Appendix S1 for further description).

### Open population model

We used an open population model to examine the response of a population that has had its age structure truncated by fishing as it gradually fills in after fishing is removed (White et al. 2013a) The population is comprised of *n* age classes, where the primary state variable is **N**_*t*_, an *n *×* *1 vector of abundance in each age class *a* at time *t*,* N*_*a,t*_. An *n × n* matrix **A** represents age‐specific survivorship, and an *n *×* *1 vector **R**_*t*_ represents larval recruitment with the abundance of new recruits in year *t*,* R*_*t*_, in the first entry and zeros elsewhere (Table 1). Note that our assumption of an open population, modeled as larval recruitment independent of local population size (as might occur if the spatial scale of larval dispersal is much greater than the spatial dimension of the MPA), also implies that recruitment is independent of harvest rate. In other words, the amount of reproduction along the coastline is the same before and after MPA implementation. The full dynamics of the open population are then

(1) $$N_{t+1}={AN}_{t}+R_{t}.$$

The matrix **A** consists of annual adult survivorship given natural mortality rate *M* and, when simulating the pre‐MPA dynamics, the age‐specific fishing mortality rate *F*_*a*_, where individuals enter the fishery at age *a*_*c*_, the age of first capture:

(2) $$A=\left[\begin{array}{c} \begin{array}{ccccc} 0 & 0 & 0 & 0 & 0\\ e^{-\left(M+F_{1}\right)} & & & & \\ & e^{-\left(M+F_{2}\right)} & & & \\ & & \ddots & & \\ & & & e^{-\left(M+F_{n-1}\right)} & 0 \end{array} \end{array}\right],$$

where *F*_*a*_ = 0 for *a < a*_*c*_ and *F*_*a*_ *= F* otherwise for the pre‐MPA dynamics, and *F*_*a*_ = 0 for the post‐MPA simulations.

**Table 1:** Symbols used in this article

Symbol	Description
**N**_**t**_	n x1 vector of abundance in each age class, a
**N**_**0**_	Initial conditions in the fished state
**R**_**t**_	n x 1 vector of recruit abundance
**A**	Population projection matrix
*n*	Number of age classes
*a*	Age class
a_c_	Age at first capture
a_m_	Age at maturity
L_fish_	Length at first capture
L_mat_	Length at maturity
L_inf_	Asymptotic maximum length
L_a_	Length at age a
*k*	von‐Bertallanfy growth parameter
a_0_	Age at length 0
*F*	Fishing mortality rate
*M*	Natural mortality rate
w_a_	Weight at age a
*p*	Weight length parameter
*w*	Weight length parameter
f_a_	Fecundity at age
θ	Angle between **N**_**0**_ and stable age distribution in the MPA
*P*	Period of oscillations
ρ	Rate of return to stable age distribution in the MPA
σ_R_	Recruitment standard deviation

To quantify trajectories in population biomass, we convert numerical abundance (***N***_*t*_) to biomass by first finding lengths at age, *L*_*a*_, using the von Bertalanffy growth equation with asymptotic maximum length $L_{\infty }$, growth rate *k*, and hypothetical age at length 0, *a*_0_:

(3) $$L_{a}=L_{\infty }\left(1-e^{-k\left(a-a_{0}\right)}\right).$$

Then we convert lengths to weights‐at‐age (*W*_*a*_) using the equation $W_{a}=pL_{a}^{w}$, where *p* and *w* are constant parameters unique for each species. Finally, we multiply weights at age by the abundances in each age class, *N*_*a,t*_, and sum over all ages to obtain biomass time *t*,* B*_*t*_.

We first analyzed deterministic expectations for the time scale and magnitude of population responses given recruitment *R*_*t*_ constant in time. We then added stochasticity to this model by assuming that larval recruitment varied randomly and independently among years, as has been observed in nearshore California rockfish species (Caselle et al. 2015, White et al. 2016). Recruitment variation is typically the largest source of interannual variability in these populations. To capture the typical boom‐bust pattern of recruitment variability, we simulated annual recruitment (*R*_***t***_) as the same constant times a draw from a lognormal distribution, e^*G*^, where *G* is a Gaussian random variable with a mean of zero and a standard deviation σ_*R*_,. Note that the value chosen for constant recruitment, 500 individuals, does not affect the magnitude of our results, which are expressed as ratios of abundances.

#### Model analysis

We first examined the differences in the biomass and abundance‐at‐age at various times as the deterministic model filled in, to illustrate the effect of the MPA on age structure of the population. We then calculated the expected deterministic trajectory of abundance and biomass after MPA implementation. Following White et al. (2013a), we express abundance at a given time *t* after implementation as a ratio relative to abundance at time 0 (i.e., the time of implementation), which we denote as $N_{t}^{\prime }/N_{0}^{\prime }$, where prime symbols indicate that we are only considering ages ≥ *a*_*c*_. White et al. (2013a) showed that the continuous time expression for the rate at which the ratio $N_{t}^{\prime }/N_{0}^{\prime }$ increases from 1 to its asymptotic maximum value depends on the exponential term *e* ^–*Mt*^ (White et al. 2013a) . This can be seen as the ratio beginning at 1 at *t *=* *0, then approaching its asymptotic maximum value, (*M *+ *F*)/*M*, as the difference between those values declines exponentially:

(4) $$\frac{N^{\prime }\left(t\right)}{N^{\prime }\left(0\right)}=\frac{\left(M+F\right)}{M}-\left[\frac{\left(M+F\right)}{M}-1\right]e^{-Mt}.$$

This expression assumes the number of age classes *n* is infinite. Note that this value is independent of the number of recruits: the number of individuals in each age class (which sum to the total population size) is a fraction of the number of recruits, specifically the proportion that survive to that age, with a different proportion surviving with and without fishing. Therefore, the number of recruits is a common factor to both $N^{\prime }\left(t\right)$ and $N^{\prime }\left(0\right)$ (White et al. 2013a, SI2). The maximum increase in abundance due to the filling in of a discrete age distribution then depends on the ratio of the annual mortality rate with and without fishing:

(5) $$\frac{N_{\infty }^{\prime }}{N_{0}^{\prime }}=\frac{1-e^{-\left(M+F\right)}}{1-e^{-M}}.$$

This expression allows us to solve for the time at which the population will reach some proportion *q* (e.g., 0.95) of its asymptotic maximum final abundance, which we can approximate using [−ln(1−*q*)/*M*]. The parallel analytic solution for the ratio of final to initial biomass is similar, but more complicated (Appendix S2).

We simulated 500 time series of the stochastic version of the model for each species. We separately simulated the dynamics in an MPA (*F *=* *0) and the dynamics if fishing continued at rate *F*. These dynamics portray the decision problem of having to determine whether abundance inside the MPA has increased relative to business‐as‐usual baseline conditions (or possibly the density at a non‐MPA reference site), with stochasticity potentially obscuring the deterministic signal. We used receiver‐operating characteristic (ROC) curves to determine how this decision problem would change over time and for different species as the population inside the MPA reached its maximum abundance using the R package pROC (Robin et al. 2011). The ROC calculations involve comparing the distribution of simulations of the MPA and reference (fished) scenarios (taking each distribution to represent the range of possible future outcomes for each scenario). These comparisons allowed us to evaluate whether abundance inside the MPA has returned to the unfished state or remains similar to the fished state, based on the probability of different outcomes through the stochastic simulations. The ROC curves are essentially a means of considering the statistical consequences of a range of possible abundance threshold values of the inside‐MPA abundance at which a manager would decide that the MPA had increased to its projected value. For each threshold we calculated the *sensitivity*, the probability of true positive decisions (the proportion of the MPA distribution greater than that threshold), and the *specificity*, which is the probability of true negative decisions (the proportion of the non‐MPA distribution less than the threshold).The area under the ROC curve (AUC) provides a single summary measure of the ability to accurately detect true positives and true negatives, in which a value of 0.5 represents there being no power to discriminate true positives and true negatives, and values closer to 1.0 indicate a high detectability of both true positives and true negatives. To understand how the rate at which decision‐making ability would increase with time for both abundance and biomass we calculated AUC at 2, 5, 10, and 20 yr after MPA implementation. To understand the life history and management factors affecting the detectability of MPA responses, we calculated correlations between the year‐10 AUC and both the recruitment variability, *σ*_*R*_, and the deterministic asymptotic abundance ratio $N_{\infty }^{\prime }/N_{0}^{\prime }$, which depends on natural and harvest mortality as expressed in Eq. (4). We selected these values because they would be expected to affect the variability and magnitude, respectively, of the population response and thus detectability. The linear models used to assess correlations met all distributional requirements.

### Closed population dynamics

To characterize the possible transient behavior of a demographically closed population, we evaluate the analogous model to Eq. (1) without external recruitment,

(6) $$N_{t+1}={AN}_{t},$$

where now the projection matrix **A** (Eq. (2)) includes reproduction given the age‐specific per‐capita fecundity *f*_*a*_ to capture internal (closed) recruitment:

(7) $$A=\left[\begin{array}{c} \begin{array}{ccccc} f_{1} & f_{2} & \ldots & f_{n-1} & f_{n}\\ e^{-\left(M+F_{0}\right)} & & & & \\ & e^{-\left(M+F_{a}\right)} & & & \\ & & \ddots & & \\ & & & e^{-\left(M+F_{a}\right)} & 0 \end{array} \end{array}\right].$$

where *F*_*a*_ is the same as in Eq. (2). We assumed that fecundity was proportional to age‐specific mass, *W*_*a*_, with proportionally constant larval survival,α. Fecundity was zero for *a < a*_*m*_, the age of maturity.

We adjusted the value of the constant α (which could be thought of as the larval survivorship) such that these populations experience self‐replacement (i.e., no increase or decrease, such that the dominant eigenvector of **A,** λ, is equal to 1) when there is no harvest. This leads to a conservative estimate given the uncertainty in the larval survival that drives uncertainty in what will occur when harvest ceases on a fished population (from a decision standpoint): the population will stop declining, but will not begin to increase geometrically (as it would for λ > 1). Over time, **N**_*t*_ will asymptotically approach a stable age distribution (SAD) given by the dominant right eigenvector **w**_1_ of **A**; in the short term after harvest ceases, **N**_*t*_ will exhibit transient behavior.

#### Model analysis

We employed three metrics to express the magnitude and duration of possible transient behavior of closed population behavior, after harvest has ceased: (1) the vector angle (θ) between initial (harvested) age vector (**N**_0_) and SAD (**w**_1_), (2) the damping ratio (ρ), and (3) the dominant period of possible transient oscillations *P* (Caswell 2001, White et al. 2013a). The first is a measure of how close the shape of the fished (initial, pre‐MPA) age structure is to the shape of the unfished (eventual, post‐MPA) stable age distribution. The definition of θ is

(8) $$\theta =\text{arc}\;\cos\frac{N_{0}\cdot w_{1}}{\left|\left|N_{0}\right|\right|\;\left|\left|w_{1}\right|\right|}$$

where double vertical bars indicate the vector norm. Essentially, it measures how much fishing has truncated the age distribution. A smaller θ indicates that the initial (fished) age distribution is closer to the unfished stable age distribution, and thus the initial population trajectory is closer to the final population trajectory.

The damping ratio, ρ, is the rate of convergence to asymptotic behavior, and is approximately proportional to the ratio of the first (λ_1_) and second (λ_2_) eigenvalues of **A**:

(9) $$\rho \approx \frac{\lambda_{1}}{\left|\lambda_{2}\right|}$$

Smaller values of ρ indicate the transient behavior will last longer, because the greatest oscillatory component of **A** (represented by λ_2_) is large relative to the asymptotic exponential growth component *(*λ_1_). The population converges on the SAD at rate *e* ^–*t* ln(ρ)^, so that 1/ln$\left(\rho \right)$ is a characteristic time scale (with units of years) for the duration of the transient.

The dominant period of transient oscillations, *P*, also depends on the oscillatory component of **A** as measured by λ_2_:

(10) $$P=2\pi /\text{arctan}\left(\frac{\text{Im}\left(\lambda_{2}\right)}{\text{Re}\left(\lambda_{2}\right)}\right),$$

where Im(*x*) and Re(*x*) denote the imaginary and real parts of a complex number, respectively. Finally, we simulated the dynamics of a population harvested at rate *F* until it reached a stable (harvested) age distribution, then ceased harvest and calculated the time it took the population to reach 95% of the stable (unharvested) age distribution.

#### Parameterization

We derived natural mortality rates (*M*) from estimates used in stock assessments or found in the literature prior to MPA implementation (Table 2). We derived fishing mortality rates (*F*) from the literature or stock assessments by averaging across years leading up to MPA implementation beginning in the 1990s to 2000s, depending on data availability (Table 2). We obtained von Bertalanffy growth parameters from (Lea et al. 1999) and fishbase.org (Froese and Pauly 2018). For the age at entry into the fishery *a*_*c*_, we converted lengths at entry to the fishery, *L*_*c*_, from stock assessments and commercial size limits to ages by inverting the von Bertalanffy equation:

(11) $$a_{c}=\frac{\ln\left[\left(L_{c}-L_{\infty }\right)/L_{\infty }\right]}{-k}+a_{0}$$

For variation in recruitment, we used model results from running a state‐space integral projection model (White et al. 2016) to hindcast recruitment for blue rockfish and black rockfish. For all other species, where sufficient data for time series‐based estimation were not available, we determined recruitment variation parameters from stock assessments (Table 2).

**Table 2:** Parameter estimates for nearshore species used in matrix projection models

Species	Max age	*L*_mat_	*L*_*c*_	*M*	*F*	*L*_*∞*_	*k*	*a*_*0*_	*a*_*m*_	*a*_*c*_	*p*	*w*	*σ*_R_	Source
Kelp rockfish	25	18.00	25.00	0.20	0.17	37.80	0.23	−0.70	3	3	6.29 × 10^−05^	3.17	0.53	Lea et al. (1999), White et al. (2016)
Blue rockfish	44	27.09	21.03	0.14	0.17	38.15	0.17	−1.15	6	4	9.77 × 10^−05^	3.09	0.78	Key et al. (2008), White et al. (2016)
Black rockfish	50	40.23	29.00	0.14	0.05	45.11	0.33	0.75	7	4	5.81 × 10^−05^	3.19	0.5	Cope, Sampson, et al. (2015)
Gopher rockfish	30	17.00	25.40	0.20	0.17	34.10	0.23	−0.50	3	6	1.30 × 10^−04^	3.08	0.5	Key et al. (2005)
Lingcod	25	49.30	60.11	0.25	0.23	96.74	0.17	−1.56	3	4	7.13 × 10^−06^	3.41	1	Hamel et al. (2009)
Copper rockfish	50	32.00	29.70	0.09	0.08	56.50	0.14	−1.00	5	5	8.98 × 10^−06^	3.13	0.8	Cope, Dick, et al. (2015)
California scorpionfish	21	17.00	25.40	0.25	0.19	40.29	0.13	−2.69	2	5	1.95 × 10^−05^	3.17	0.5	Monk et al. (2017)
Brown rockfish	34	27.50	27.50	0.14	0.13	51.40	0.16	−0.55	4	4	1.02 × 10^−05^	3.07	0.8	Cope, Dick, et al. (2015)
Yellowtail rockfish	64	37.00	28.50	0.11	0.06	49.88	0.18	−1.21	6	4	6.69 × 10^−05^	3.00	0.8	Cope, Dick, et al. (2015)
Vermillion rockfish	65	36.00	22.00	0.10	0.14	53.92	0.16	−0.18	7	3	1.46 × 10^−04^	3.04	0.7	MacCall (2005)
Bocaccio	55	35.50	38.00	0.15	0.01	70.00	0.22	−0.69	3	3	1.32 × 10^−05^	3.00	1	He and Field (2017)
Cabezon	17	34.00	38.10	0.28	0.12	49.90	0.28	−1.23	3	4	5.50 × 10^−06^	3.19	1	Cope and Key (2009)
China rockfish	83	27.00	30.48	0.06	0.09	33.62	0.23	−0.26	7	10	7.79 × 10^−05^	3.18	0.8	Cope, Dick, et al. (2015)
Kelp greenling	25	30.00	30.48	0.30	0.17	41.15	0.24	−1.91	3	4	4.18 × 10^−05^	3.00	0.45	Berger et al. (2015)
California sheephead	53	24.00	30.48	0.25	0.25	46.70	0.18	1.00	4	6	2.89 × 10^−05^	2.86	0.61	Alonzo et al. (2004)
Red sea urchin	100	18	20	0.07	0.36	33.15	0.23	−0.7	3	6	1.27 × 10^−03^	2.71	0.5	Morgan et al. (2000)
Kelp bass	33	22.3	30.48	0.18	0.12	69.8	0.06	−3.5	3	6	2.73 × 10^−06^	3.27	0.5	Love et al. (1996), Jarvis et al. (2014)
Olive rockfish	30	27	30.48	0.14	0.07	33.62	0.23	−0.26	4	3	1.08 × 10^−05^	2.968	0.5	Cope, Dick, et al. (2015)*
Black & Yellow rockfish	30	17.5	24	0.2	0.17	24.95	0.23	−0.38	5	14	1.12 × 10^−04^	3.114	0.5	Key et al. (2005)*

Model implementation used R statistical programming language (R Development Core 2018), and code is available for download online through the R package (see Data Availability statement).

## Results

To present the results of these analyses for our 19 modeled species, we show summary metrics across all species along with detailed trajectories for four example species expected to show a range of responses: (1) bocaccio, a species with low pre‐MPA fishing mortality rates relative to natural mortality; (2) blue rockfish, a commonly fished rockfish with moderate pre‐MPA fishing mortality rates; (3) China rockfish, a long‐lived rockfish with moderate pre‐MPA fishing mortality rates; (4) red sea urchin, a long‐lived invertebrate with a high pre‐MPA fishing mortality rate. Detailed results for the remaining 15 species are presented in the appendices, as indicated.

### Open population dynamics

**Figure 1:**
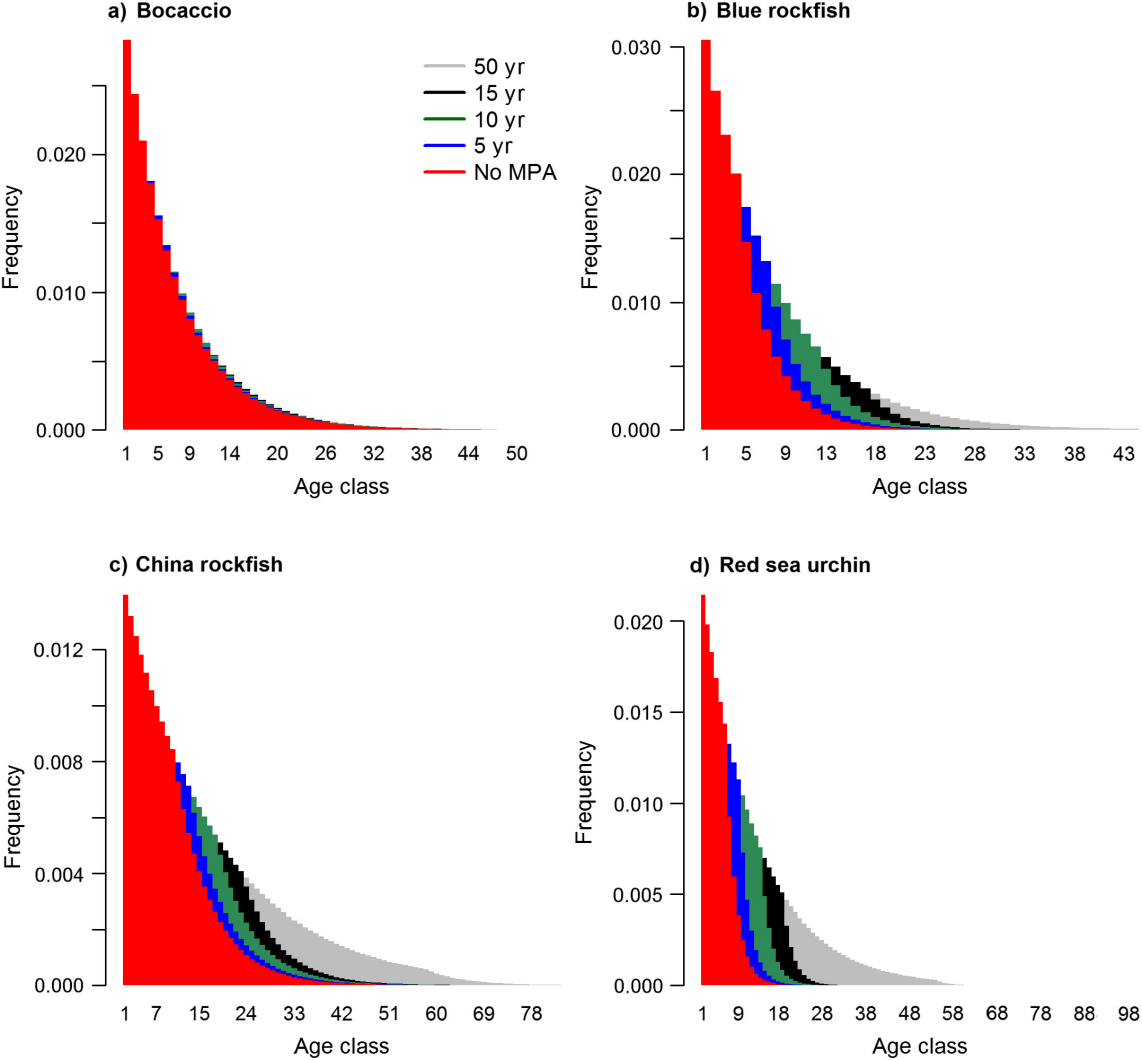
Age distribution of nearshore fished species before and after marine protected area (MPA) implementation. Red shading indicates the fished population at equilibrium; the remaining colors denote a previously fished population in the first 5–50 yr of protection inside an MPA. Results shown for (a) bocaccio, (b) blue rockfish, (c) China rockfish, and (d) red sea urchin. Simulations assume open population dynamics with deterministic recruitment.

In our four example species, time sequences of the filling‐in process for age distributions illustrate the differences between species in magnitude and timing (Fig. 1; Appendix S3: Fig. S1; note that the age scale of the ordinates differ, as do the ages at which fishing begins). The most obvious difference among the species is the small expected increase for bocaccio relative to other species, because our parameterization has bocaccio at a low fishing rate prior to protection based on current stock assessments (this may not be the actual age structure because the stock was heavily exploited prior to 1999, when severe harvest restrictions were imposed). In all of these figures, there is a “front” (starting with the red distributions in Fig. 1) that declines to the right from age *a*_*c*_ at the pre‐MPA mortality rate (e^‐(*M *+ *F*)^), corresponding to the harvested age distribution. These fronts move to increasing ages (to the right), shown here in 5‐yr increments, until they fill in the unfished age structure (which is declining to the right from age zero as e^−*M*^).

**Figure 2:**
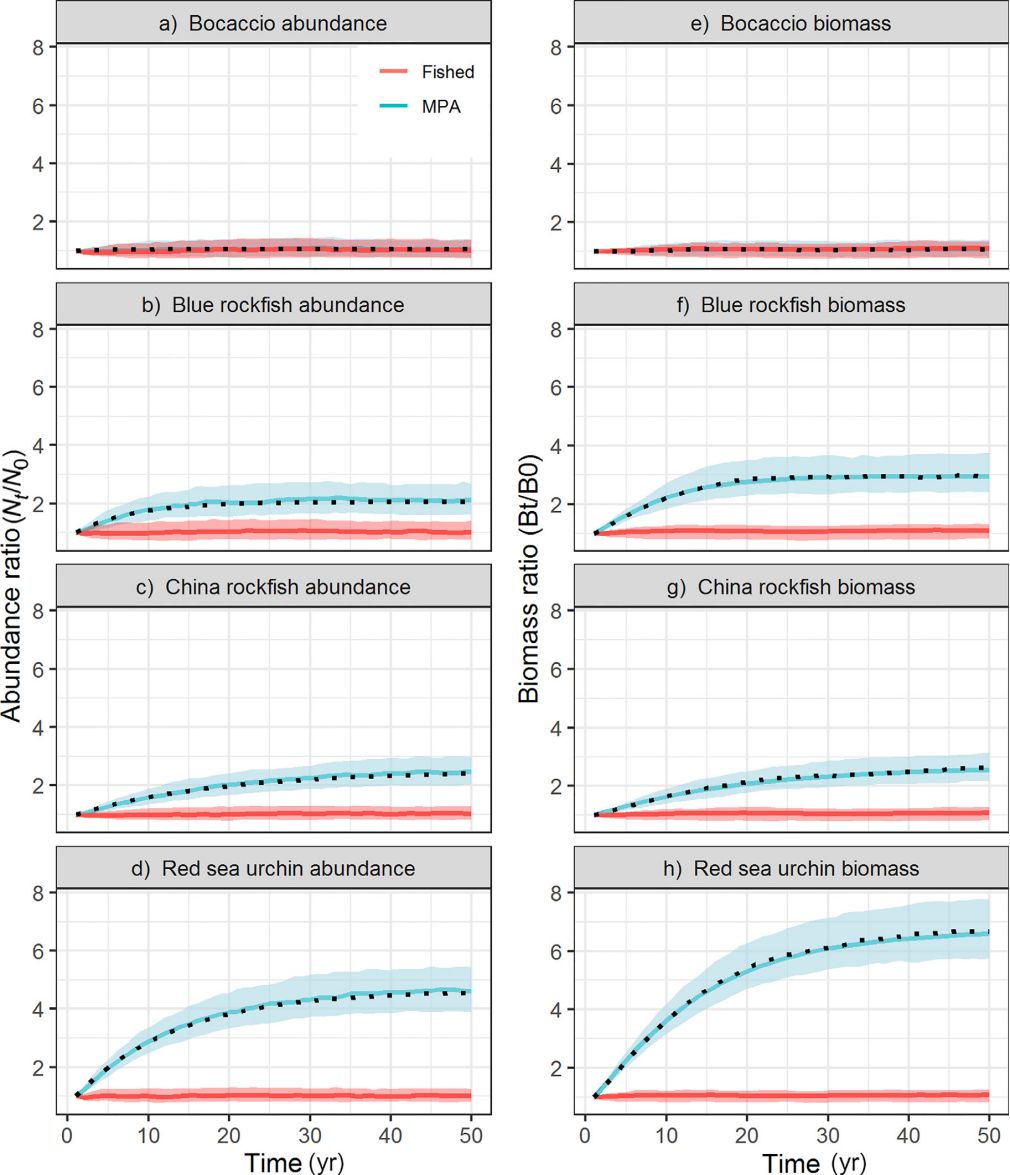
Trajectories of (a–d) population abundance and (e–h) biomass ratios over time, with (blue) and without (red) MPA establishment, given open population dynamics with stochastic recruitment. Colored bands represent the lower quartile and upper quartile for 500 simulated runs, and solid colored lines indicate median responses. Greater separation between the ranges of outcomes with MPA vs. without MPA establishment indicates greater ability to distinguish an MPA effect. Black dotted lines represent deterministic model projections inside an MPA. Black tick mark on left side of abundance plots indicates analytic result of final abundance ratio increase.

**Figure 3:**
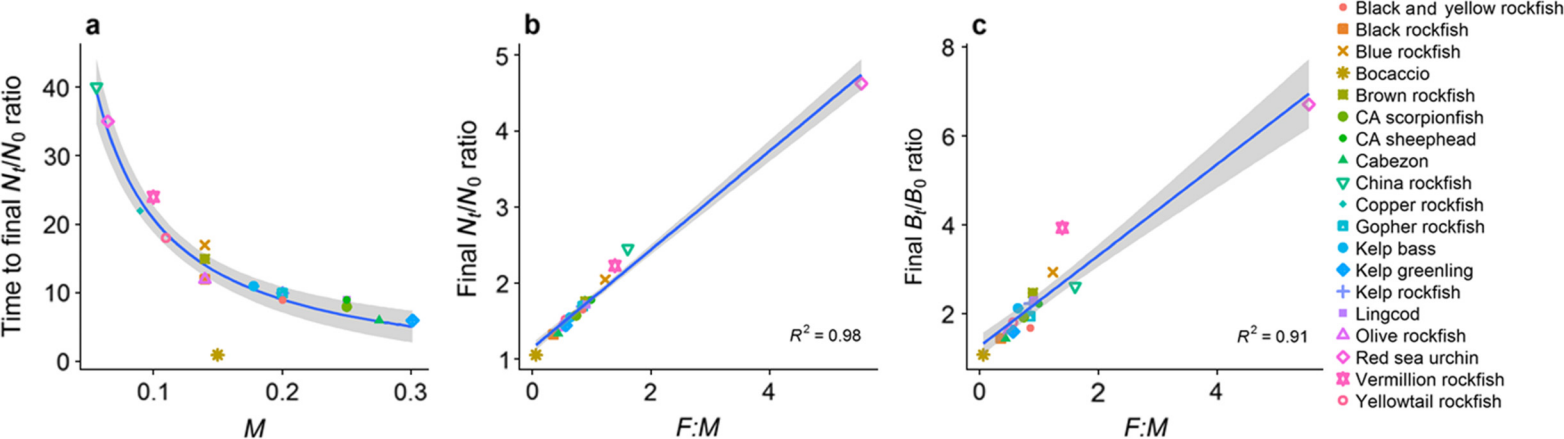
Relationship between trajectories of open populations inside MPAs and demographic parameters. Panels show the relationship between (a) time to reach 95% of the final abundance ratio and the natural mortality rate, *M*, and between (b) final abundance ratio and (c) final biomass ratio and the ratio of fishing mortality rate to natural mortality rate (*F*:*M*). Linear model with 1/*M* transformation for the x axis in panel a and gray bands represent 95% confidence interval around predictions from linear models. Note different *y*‐axis values in panels b and c. Values based on deterministic simulations with open population dynamics.

The population trajectories initially increase rapidly then more slowly to asymptotically reach a maximum for each species (thick curves in Figs. 2 and S2; plotted as the ratio of current abundance to original fished abundance $N_{t}^{\prime }/N_{0}^{\prime }$). The approximation of Eq. (4), (*M *+ *F*)/*M *=* *1 + *F*/*M* (White et al. 2013a) accurately represents this maximum and therefore provides a useful means for quickly calculating the expected final ratio due to filling in. Accordingly, the *F*/*M* ratio closely predicts the final abundance and biomass ratios for all 19 species (*R* ^2^ = 0.98 and 0.91, respectively; excluding red sea urchin, which is a high‐leverage point along the abscissa, we obtain *R* ^2^ = 0.98 and 0.73 respectively, Fig. 3b, c). The increase in biomass (up to 7‐fold) is always greater than the increase in numerical abundance (up to 4.5‐fold) because it accounts for the increase in mass with age in addition to the increase in abundance (Figs. 2, 3a, b, Appendix S2).

Deterministically, the time to reach the full benefit of filling in ranges from 1 yr for bocaccio to almost 40 yr for China rockfish (Fig. 3a, Appendix S3: Table S1, calculated from simulations as the time to 95% of the maximum abundance). Because abundance increases from the fished abundance to the unfished abundance as 1−*e* ^−*Mt*^, where *t* is the time since MPA implementation, a suitable approximation for the time to 95% is −ln(0.05)/*M* (Appendix S3: Table S1). As such, 1/*M* is a strong predictor of the time scale of responses across all species (Fig. 3a).

**Figure 4:**
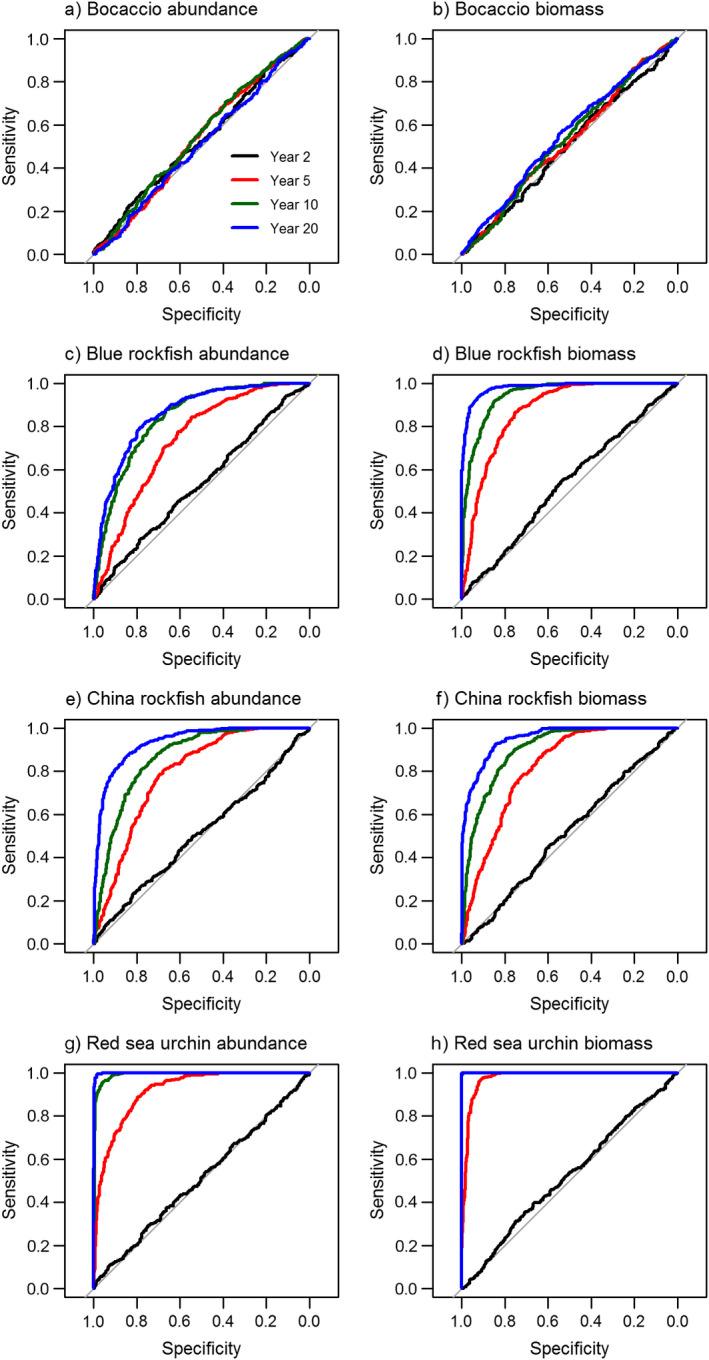
(a, c, e, g) Abundance and (b, d, f, h) biomass ratio changes receiver‐operating characteristic (ROC) curves in an MPA compared to fished state for 2 yr, 5 yr, 10 yr, and 20 yr after MPA implementation for four example species (rows). Sensitivity measures the likelihood of detecting true positives, and specificity measures the likelihood of detecting true negatives. As the fished and unfished distributions in Fig. 2 diverge from each other, the probability of detecting a true positive increases, and the probability of detecting a false positive declines. Simulations assume open population dynamics with stochastic recruitment.

The capacity for stochasticity to obscure responses and increase the delay to a detectable response is illustrated by the degree of separation between the shaded regions in Fig. 2; Appendix S3: Figs. S3, S4. They indicate that (1) evaluation (i.e., assessment of whether or not an MPA is working as expected) will be informative earlier using biomass rather than abundance, (2) of the indicators and four sample species, biomass of red sea urchin have the greatest difference between fished and unfished distributions and therefore likely serve as the most reliable indicator of MPA efficacy, and (3) bocaccio (as modeled here, not using current age structure) is unlikely to be useful as an indicator of MPA success. Considering not just the quartiles as illustrated in Fig. 2, but the full distributions that underlie the probabilities of detecting both true positives and true negatives in the ROC curves in Fig. 4, evaluation skill (i.e., high probability of a true positive, and low probability of a false positive) generally increases with time (after 2, 5, 10, and 20 yr). Of the four example species, the earliest detection (i.e., the first time the ROC curve bends away from the diagonal) occurs for red sea urchin (Fig. 4e, f), likely due to intense harvest and fast growth in that species. In contrast, bocaccio, the species with the fastest deterministic response, never exhibits a detectable response given stochastic recruitment (Fig. 4a, b) due to the low pre‐MPA harvest and therefore low magnitude of response overall.

**Figure 5:**
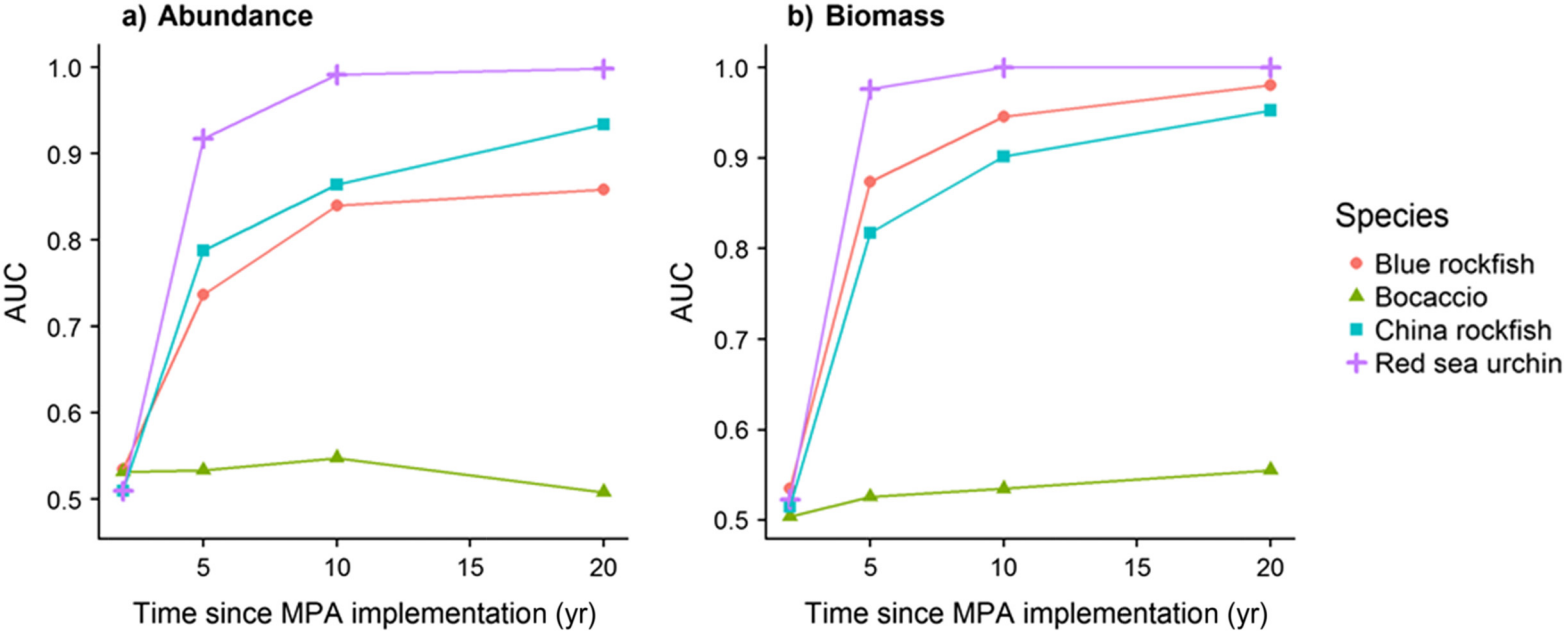
MPA response detectability for representative species, measured as area under the ROC curve (AUC) for different species at different time points since MPA implementation for (a) changes in abundance and (b) biomass in the MPA as compared to the fished state. Simulations assume open population dynamics with stochastic recruitment.

**Figure 6:**
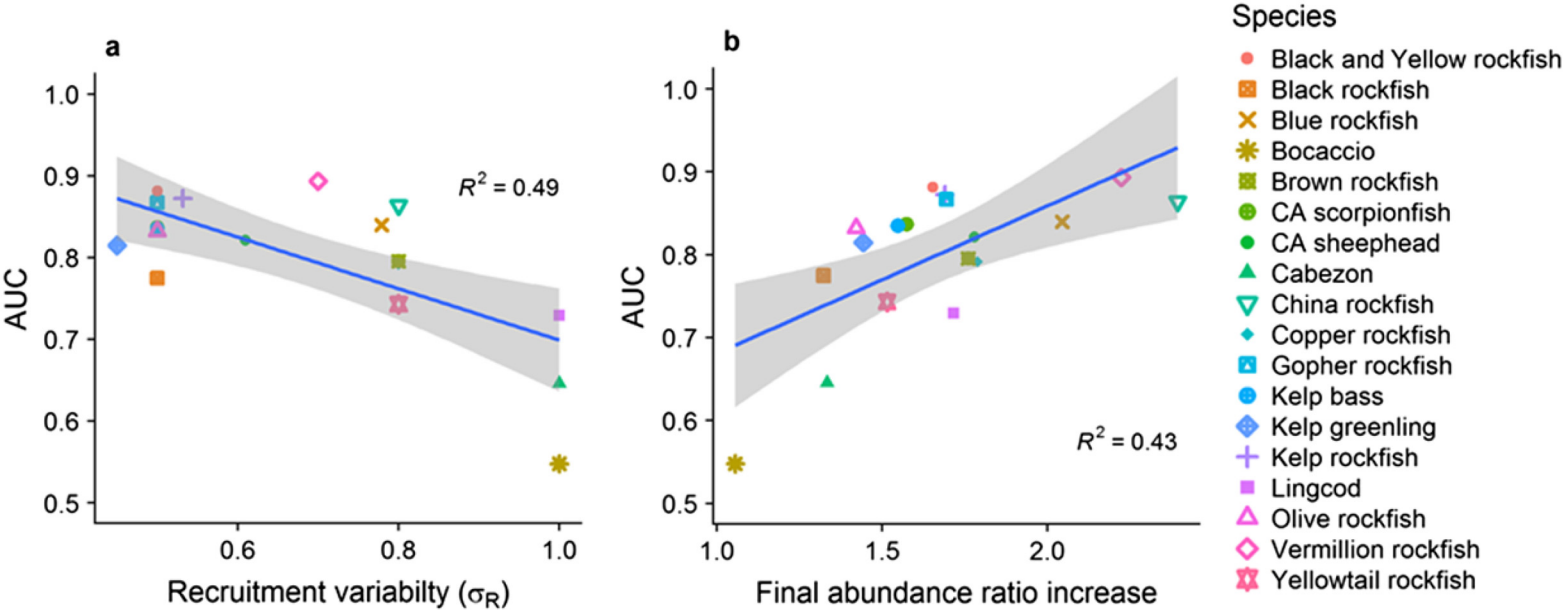
Relationships between MPA response detectability (measured as area under the ROC curve [AUC] for abundance 10 yr after MPA implementation) and (a) recruitment variability (*σ*_R_) and (b) final abundance ratio increase (*N*_*t*_/*N*_0_ = (*M *+ *F*)/*M*), excluding red sea urchins *R* ^2^ = 0.43. Gray bands represent 95% confidence intervals from linear regression. Simulations assume open population dynamics with stochastic recruitment.

In general, across all 19 species, greater detectability of an MPA response (greater AUC, or area under the ROC curves, which integrates over both sensitivity and specificity) occurred for biomass rather than with abundance due to the greater magnitude of biomass response (Fig. 5, Appendix S3: Fig. S5). Detectability in both metrics began to saturate around 10 yr for most species (Fig. 5, Appendix S3: Fig. S5). Consistent with intuition, species with greater recruitment variability had lower AUC at 10 yr *R* ^2^ = 0.5 (Fig. 6a), and species with greater final abundance ratio (*M *+ *F*)/*M* had greater AUC at 10 yr *R* ^2^ = 0.44, excluding red sea urchins *R* ^2^ = 0.43 (Fig. 6b). Detection power may saturate as AUC approaches 1 for species with high final abundance ratio increases (Fig. 6b).

To inform expectations for the probability of a detectable response beyond our study system, we derived an analytical expression for the spread around the (*M *+ *F*) /*M* expectation due to recruitment variability. The variance of the ratio of summed abundances $N_{t}^{\prime }/N_{0}^{\prime }$, will gradually increase as new recruits arrive and the age‐structure fills in during the transient period, but the variance introduced at the recruit stage (age 1) will be dampened over time by natural mortality (for details of derivation see Appendix S4). Eventually, the standard deviation (σ_*total*_) of abundance for the sum of the fished age classes at time *t* after MPA implementation will be:

(12) $$\sigma_{\mathrm{total}(t)}={\left[\sum_{a=0}^{t}{(\sigma_{R}e^{-M\left(a+a_{c}\right)})}^{2}\right]}^{0.5}$$

where σ_*R*_ is the standard deviation of recruitment. Thus variability in the population response to MPAs depends both on variability in larval recruitment and the natural mortality rate.

### Closed population dynamics

**Figure 7:**
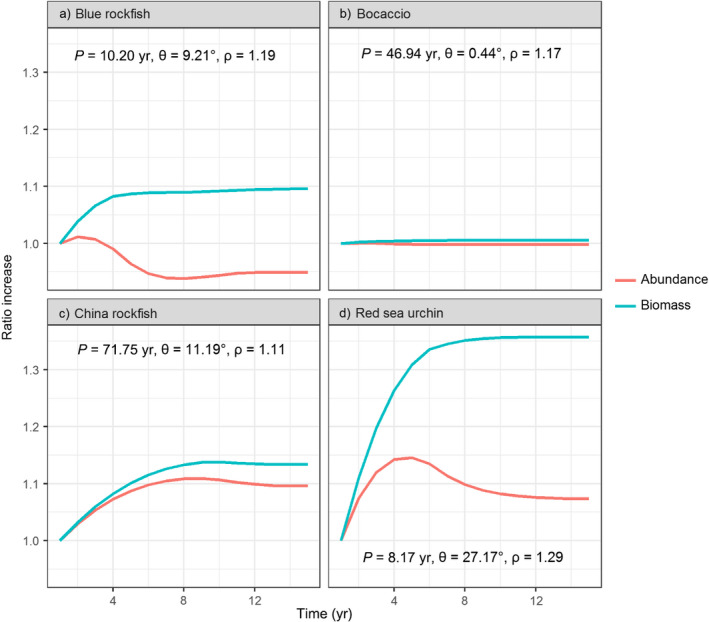
Closed, deterministic population responses in an MPA for abundance (red) and biomass (blue), plotted as ratios to their initial (fished) values, for four example species (panels). Inset values indicate the period of oscillations in years (*P*), the similarity to stable age distribution (θ), and the rate of convergence to asymptotic behavior (ρ).

**Figure 8:**
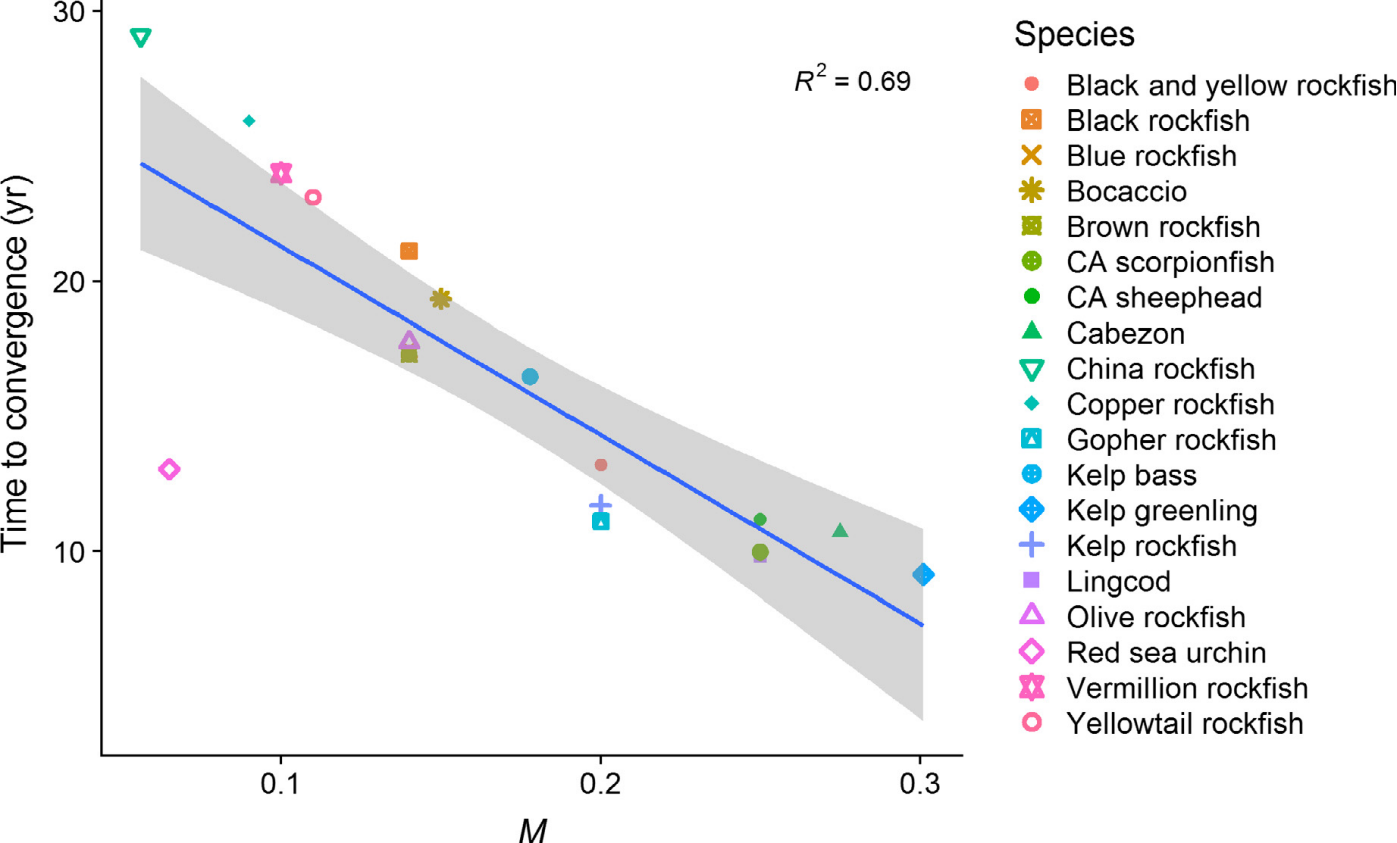
Relationship between closed population time to convergence, 1/log(ρ), which is inversely related to the natural mortality (*M*). Gray bands represent 95% confidence intervals from linear regression.

**Figure 9:**
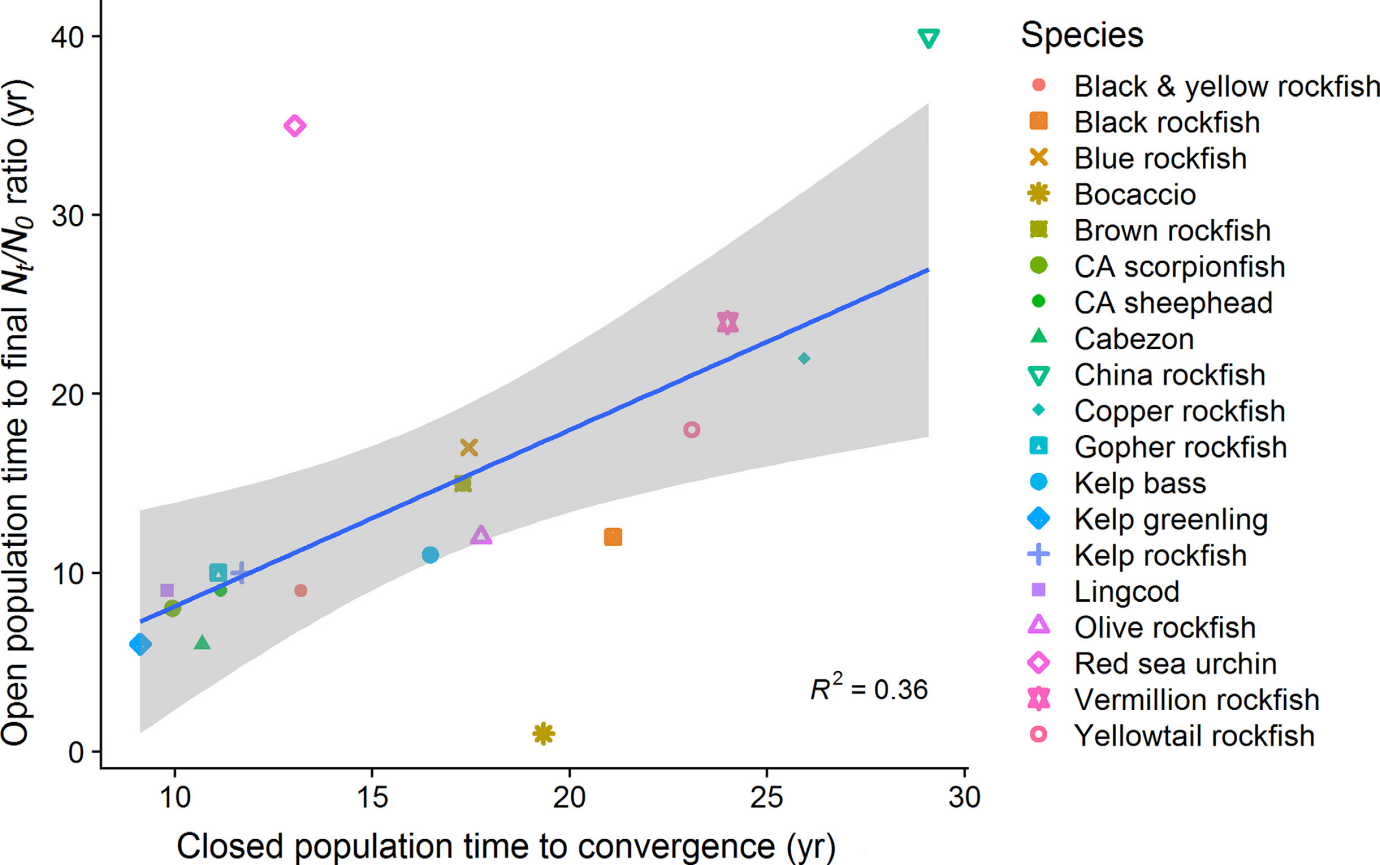
Relationship between the closed population transient, time to convergence measured by 1/log(ρ), and the open population transient time to reach 95% of the final abundance ratio.

In closed populations, where reproduction from filled‐in age classes can further contribute to abundance increases, oscillations in initial population responses can occur in some species (e.g., Figs 7a, c, d). However, oscillations are typically small (as measured by the angle between fished and unfished age structures θ (Appendix S3: Table S2), and in terms of the amplitude of oscillations relative to the magnitude of post‐MPA increase in abundance in the open population model [Fig. 2]) and dampen out within a few years (as measured by the inverse of the damping ratio ρ and the dominant period of oscillations *P*; Fig. 7; Appendix S3: Table S2) for the suite of species in our model system. The greatest values for expected oscillations and transients occurred for red sea urchin (θ) and China rockfish (*P*, 1/log(ρ), Fig. 7d; Appendix S3: Table S2) due to intensive fishing early in the age structure for these longer‐lived species. Longer duration of transient behavior in the closed populations, as measured by 1/log(ρ), the time scale of convergence, occurs with characteristics that lead to slower life histories such as lower natural mortality (Fig. 8). Note that other life history factors such as the coefficient of variation in the spawning age structure also drive transient dynamics in the closed population model (Taylor 1979, Caswell 2001); here we focus on the relationship with natural mortality for comparison to the open population model (Fig. 3a). The length of the transient period between the open and closed population models had a weak positive correlation (*R* ^2^ = 0.36, Fig. 9), which reflects the fact that life history and prior fishing affect the length of transient dynamics under these two connectivity assumptions in different ways. We place more emphasis on relative values and drivers of the time to convergence in the closed population model because the absolute values depend on our assumption of calibrating the poorly known larval survivorship values such that λ_1_ = 1 in the unfished state. This conservative assumption causes earlier time to convergence and smaller increases in abundance and biomass in the closed population model relative to the open population model.

We also assessed stochasticity in the closed population case (Appendix S5). In contrast to the open population model, in which statistical detectability generally increased over time, detectability in the closed population model had the potential to decrease over time. This difference arose because variability enters the open model as an additive random variable, so that population abundance eventually achieves a constant variance, and detectability improves as the deterministic signal grows larger than that variance. In the closed model, stochasticity enters multiplicatively because it modifies the geometric growth rate of a linear population model, causing the variance in the distribution of population abundances to grow over time.

## Discussion

Here, we have shown how the filling in of the age structure of fished populations responding to MPAs can increase abundance and biomass by as much as seven‐fold over a period as long as forty years. Moreover, these trajectories can be predicted from life history and management factors, primarily natural mortality *M* and pre‐MPA fishing mortality *F*, allowing us to set expectations for adaptive management. Previous efforts to understand the effect of prior management on population responses to MPAs have simply divided species into harvested and non‐harvested categories, and have found that empirical observations also support harvest rate as a primary predictor of MPA response (Micheli et al. 2004, Hamilton et al. 2010, Caselle et al. 2015). Here, we have shown that different harvested species within the same MPA will respond at different time scales and that the response will be an asymptotic approach to a maximum (regardless of the assumption about open or closed dynamics). This result suggests that there may not be a linear or exponential increase in abundance or biomass following MPA implementation, which may explain the conflicting results from meta‐analyses for whether population responses increase with MPA age, typically tested using linear regressions (Côté et al. 2001, Halpern and Warner 2002, Claudet et al. 2008, Molloy et al. 2009, Vandeperre et al. 2011). While the importance of harvest and natural mortality match intuitive expectations, they are also typically poorly known parameter values. Therefore, our results reinforce the need to measure these values in order to develop quantitative expectations for the magnitude and time scale of MPA responses in an adaptive management framework.

Our projections provide context for, and are in line with, observed responses in California's MPAs. In central California, no species showed detectable increases in biomass within MPAs in the 7 yr since establishment, with high temporal variability (Starr et al. 2015), in line with our expectations of at least 10 yr to detectable responses for most species. However, in the southern Californian Channel Islands, significant biomass increases did occur within 5 yr of MPA establishment (Hamilton et al. 2010) and continued through 9 yr since establishment (Caselle et al. 2015). One potential explanation for the difference in detectable responses between locations is differences in temporal recruitment patterns. MPA establishment in central California was followed by several years of poor recruitment (Starr et al. 2015) while there were several years of favorable recruitment following MPA establishment in the Channel Islands (Caselle et al. 2015). Note that Caselle et al. (2015) did not find significant increases in the Channel Islands location furthest from port and therefore with the lowest expected fishing, in line with our expectations for the predominant role of fishing mortality. While the detectability of the Channel Islands responses are earlier than might be expected from our projections, the magnitudes are in line with our expectations: the confidence intervals around observed responses (two‐fold increases in densities of lingcod, blue rockfish, California sheephead; approximately 1.5‐fold increases in densities of gopher rockfish, olive rockfish, vermilion rockfish, and copper rockfish; slightly lower increases for cabezon and kelp bass; Hamilton et al. 2010) are consistent with our confidence bounds from stochastic simulations. Our predictions suggest further expected increases beyond those observed in the Channel Islands. For example, for blue rockfish we predict a nearly three‐fold increase in biomass 21 yr after MPA implementation, while vermilion rockfish is projected to increase nearly four‐fold in 31 yr.

Placing observed responses in the context of our model expectations provides a quantitative test of whether hypothesized drivers (e.g., recruitment variability in time, pre‐MPA harvest variability in space) can explain observations, or whether further explanation, in terms of either management or the natural processes, is necessary. For example, our models quantitatively validate the hypothesis that differences in recruitment can explain the differences in responses between the Channel Islands and central California. Therefore, the lack of detectable responses in less than 10 yr in central California, even though such a response did occur elsewhere, does not necessarily indicate a change in MPA design or enforcement is necessary to achieve longer‐term management goals. In other words, our analysis illustrates the ability for model projections to elucidate the time scales for deciding when further monitoring or management action might be necessary in an adaptive management process.

### Open vs. closed population dynamics

Our analysis of open and closed populations represent two extremes on a continuum of the spatial scale of larval dispersal. Most populations will fall in the middle, with some combination of locally retained and dispersed recruits. A key difference between the open and closed population responses is that in the closed population case there is potential for oscillatory dynamics and a negative initial trajectory (White et al. 2013a). However, we found initially negative and oscillatory responses to be relatively minor, if present, in the 19 species in our study system. In addition, the magnitude of both open and closed population responses had analogous life history predictors (e.g., natural mortality rate).

The difference between the closed and open trajectories here was the difference in rates of exponential decay to the asymptotic or steady‐state conditions, respectively, (Eqs. (4) and (9)). This difference arises because in the open population the exponential decay depends directly on natural mortality *M*, while in the closed population, the exponential decay has a more complex dependency on a wider array of life history processes (see Caswell 2001: Section 4.7). Note that the open model did not include a potential coast‐wide increase in recruitment due to MPA effects, where whether such an increase might occur will likely depend on additional factors such as whether MPAs reduce or displace fishing effort. Which time scale more accurately represents a given population depends on the scale of dispersal and the size of the MPA. White et al. (2013a) showed that when the fraction of self‐recruitment is relatively low (<30%), as would be expected for species with longer larval dispersal distances relative to MPA size) then population responses are similar to the open population case. However, when the fraction of self‐recruitment is high (≥30%, as would occur for species with short larval dispersal distances relative to MPA size), then the trajectory is more similar to the closed population case. For most of the species modeled in this study, the open population case likely more accurately reflect population dynamics because they have long larval dispersal distances relative to MPA size and reside in a region with fast, advective currents (Buonaccorsi et al. 2005, Shanks 2009). However, self‐recruitment rates greater than 30% have been reported for various coral reef species (Swearer et al. 1999, Almany et al. 2007) and closed population dynamics may be more relevant to MPAs in those systems.

A second difference between the open and closed cases was the difference in the effect of stochastic variability in larval recruitment. The open population eventually reached a stationary distribution of population trajectories, allowing detection skill (AUC) to increase over time, while the closed population had monotonically increasing variance in population trajectories, reducing detection skill over time. These effects are to be expected from the nature of the two models (variability entering as a linear subsidy term vs. entering as a multiplier to the growth rate). In both cases, but particularly in the closed population case, the problem of stochastic variability could be reduced in an adaptive management context by frequent monitoring. This would allow managers to update their knowledge of the actual (stochastic) trajectory, rather than knowing only that it should lie somewhere within a broad and potentially widening distribution.

### Indicators for MPA monitoring

Our detectability (AUC) results can be applied by an MPA manager seeking reliable indicator species by using this analysis to evaluate levels of confidence in determining population responses to MPAs. For example, a detectable response within 10 yr after MPA implementation (AUC > 0.8, similar to an 80% statistical power), which was the typical outcome for most species analyzed here, occurred for species with at least a two‐fold increase in and with σ_*R*_ of 0.7 or less. Species beyond those thresholds (i.e., lower magnitude of increase, higher recruitment variability) are not likely to be reliable indicators of MPA performance even if they are harvested species that can benefit from MPAs. When considering these drivers of detectability together, recruitment will be less influential on longer‐lived species with lower natural mortality, as seen by the increase in detectability as the ratio of *F/M* increases (Fig. 6).

Intuitively, our analyses demonstrate that longer‐lived species (i.e., those with lower natural mortality rates) with higher pre‐MPA harvest rates are expected to have a greater response to MPAs, but these responses will emerge over longer time scales compared to species with shorter lifespans and lower pre‐MPA harvest. Therefore, longer‐lived species with high pre‐MPA harvest rates and low recruitment variability could serve as reliable indicator species for long‐term MPA monitoring and adaptive management, allowing for the “filling in” effect to be observed over the years following MPA implementation. For example, red sea urchins will likely exhibit strong responses to protection, making this species a more reliable choice for detecting a response in monitoring, whereas no response is expected to be observed in bocaccio rockfish due to its lower pre‐MPA harvest rate, making this species an unreliable indicator of MPA efficacy on its own. Additionally, this species has been in a rebuilding phase since being overexploited in the late 1990s, so it would be difficult to distinguish MPA effects from the long‐term trajectory due to conventional management. Choosing a variety of species that differ in their expected population responses to MPAs can serve as an indication of MPA functionality. For example, choosing species with a rapid, but lower overall magnitude in response can provide initial insight into MPA efficacy, while choosing a species with a low expected response can serve as a control to determine whether or not the MPA is driving any observed increases in species that experience high fishing before MPA establishment.

For most species assessed, population responses to MPAs in terms of changes in biomass ratios are greater as compared to abundance, and the detectability of biomass is also greater at shorter time scales than for abundance. In the extreme, species with a low magnitude of response due to low pre‐MPA harvest or high variability in recruitment only have detectable biomass and not abundance responses to MPAs. The discrepancy between biomass and abundance responses occurs due to the “filling in” effect in which the largest individuals at the oldest age classes experience reduced mortality from fishing and contribute higher levels of overall biomass as compared to the biomass contribution from smaller, younger individuals. While greater in eventual magnitude and initial detectability, the time to reach those final biomass ratios were also longer than for abundance. Note also that assessing biomass requires additional data collection, and, because the biomass response is cube of the length, the error will also be cubed resulting in higher error as compared to abundance. Furthermore, in some cases the abundance response can initially exceed the biomass response, depending on the size at entry to the fishery (before MPA establishment) relative to the growth curve. Specifically, if the size at entry to the fishery is near the asymptotic region of the growth curve, then the filling in of size classes will be relatively small and therefore the primary response will be in abundance. Overall, abundance and biomass serve as complementary indices for monitoring and adaptive management, with biomass as the more reliable indicator of initial MPA effect and for species fished at smaller sizes (so long as estimation errors are carefully accounted for), and abundance as the more reliable indicator of when long‐term saturation occurs and for species fished at larger sizes.

### Modeling considerations

As with any model, the models presented here simplify a number of factors known to affect population responses to MPAs. For example, we assume natural mortality does not vary with age or size when in reality it is likely to be age‐ and size‐dependent (Miller and Hyun 2017). Such age‐ and size‐dependent mortality likely further increases the nonlinearity of population responses to MPA establishment over time, extending the time required for filling in and delaying the time to reach asymptotic growth in a closed population. We also assume that the initial age distribution is at the expected stable age distribution in the fished state, which it can depart from due to environmental stochasticity (elsewhere we quantify the effect of such departures on monitoring expectations; Nickols et al. 2019). Furthermore, we ignore density dependence, which can affect population responses to marine reserves (Lizaso et al. 2000, Gerber et al. 2003, Abesamis and Russ 2005). Negative density dependence is unlikely to affect initial responses if high fishing led to low densities, though positive (also known as inverse) density dependence or Allee effects may slow or impede populations fished to low levels (Gascoigne and Lipcius 2004, Stoner et al. 2012). Negative density dependence may also reduce longer term population responses (Abesamis and Russ 2005). We also have predicted single species responses to protection in an MPA and ignored species interactions. Negative interactions (e.g., competition, predation) can lead to declines following reserve establishment if strong enough relative to harvest (Micheli et al. 2004, Baskett and Barnett 2015, Villegas‐Ríos et al. 2017), while positive interactions (e.g., interspecific facilitation, recovery of foundational species from habitat‐damaging fishing gear) can lead to increases greater than predicted here (reviewed by Baskett and Barnett 2015).

Our analysis assumes that individuals largely stay within MPAs and have not modeled movement of fish and invertebrates across MPA boundaries, where they would be subject to harvest pressure. Most of the species in this study for which we have movement data have been found to show high site fidelity (reviewed by Freiwald 2012), so they are likely to exhibit the responses we have modeled here. However, movement outside of MPAs can lead to exposure to fishing mortality, which might be elevated if MPAs displace effort to areas outside or if fishing effort is concentrated on MPA boundaries (Kellner et al. 2007). Moffitt et al. (2009, 2013) have shown how movement of adults beyond MPA boundaries can be accounted for in an adaptive management context.

Additionally, we assume a single, constant harvest rate, taken from statewide stock assessments, when we compare ratio changes inside a MPA relative to a projected fished steady state. In reality, harvest varies in both space and time. MPAs can cause fishing to vary over time for outside‐MPA reference sites by either increasing harvest through displacement or decreasing harvest through reduced overall fishery accessibility (Roberts et al. 2001, Chollett et al. 2016, Cabral et al. 2017). Therefore, our “fished” state is more representative of before‐MPA conditions than outside‐MPA conditions. However, such before‐MPA conditions will depend on spatial variation in fishing mortality rates (e.g., as might depend on distance from ports; (Caddy and Carocci 1999). Given the central role of fishing mortality to biomass and abundance responses to protection and their timing illustrated here, estimating local fishing mortality rates (when possible) can provide a more spatially explicit expectation for the response to marine reserves at particular locations (White et al. 2016, L. Yamane, et al. *unpublished manuscript*).

The usefulness of these model results inevitably depends on the uncertainty in parameter values. As noted above, two of the most important parameters in quantifying expected species’ responses to reserves are also two of the most uncertain: local fishing (*F*) and natural (*M*) mortality rates (but we are fortunate to know this dependence). White et al. (2016) provide a method for estimating of the local value of *F* through fitting models to size distributions. Fishery stock assessments address the uncertainty in *M* by employing decision analyses that test the effects of different trial values of *M* on outcomes. Another approach is to estimate the value of *M* based on its dependence on other factors such as life history characteristics (in particular, von Bertalanffy growth parameters *L*_*∞*_ or *K*) and temperature, with two alternatives to this approach detailed in (Pauly 1980, Gislason et al. 2010).

## Conclusions

Overall, our framework informs the expected magnitude (1–7 fold), time scale (1–40 yr; with assessment of MPA efficacy typically requiring at least 10 yr), indicator metrics (biomass favored over abundance), and indicator species of responses to California MPAs. In addition, the qualitative relationships found here (e.g., increasing magnitude and detectability of response with greater ratios of fishing : natural mortality, decreasing detectability with increasing recruitment variability) can inform selection of indicators in, and expectations for detectable responses to, MPAs California. Overall, our approach provides a broadly applicable, quantitatively rigorous framework for developing the magnitude, timing, and detectability of expected responses to MPAs against which to compare observed outcomes from monitoring data in an adaptive management approach (Walters and Holling 1990, White et al. 2011).

## Data Availability

R package is available on Zenodo: https://doi.org/10.5281/zenodo.3228863
