# Supplementary material for: Setting expected timelines of fished population recovery for the adaptive management of a marine protected area network
Source: Ecol Appl. 2019 Jul 26;29(6):e01949. doi: 10.1002/eap.1949 (PMC9285580; doi:10.1002/eap.1949)
Supplement: Supplementary file 1 [file EAP-29-e01949-s005.pdf]

**Supporting Information.** Katherine A. Kaplan, Lauren Yamane, Louis W. Botsford, Marissa L. Baskett, Alan Hastings, Sara Worden, J. Wilson White. 2019. Setting expected timelines of fished population recovery for the adaptive management of a marine protected area network. *Ecological Applications.*

Appendix S1. Fishery status of 19 species assessed in this study.

In this study we assessed 12 species of rockfish, genus *Sebastes* (Scorpaenidae): kelp rockfish (*S. atrovirens*), blue rockfish (*S. mystinus*), black rockfish (*S. melanops*), gopher rockfish (*S. carnatus*), brown rockfish (*S. auriculatus*), copper rockfish (*S. caurinus*), yellowtail rockfish (*S. flavidus*), vermillion rockfish (*S. miniatus*), bocaccio (*S. paucispinis*), China rockfish (*S. nebulosus*), black and yellow rockfish (*S. chrysomelas*), and olive rockfish *(S. serranoides*); two species of Hexagrammidae: lingcod (*Ophiodon elongates*) and kelp greenling (*Hexagrammos decagrammus*); as well as California scorpionfish (*Scorpaena guttata*; Scorpaenidae); cabezon (*Scorpaenichthys marmoratus*; Cottidae); California sheephead (*Semicossyphus pulcher*; Labridae); kelp bass (*Paralabrax clathratus*; Serranidae); and an invertebrate, red sea urchin (*Mesocentrotus franciscanus*).

Kelp rockfish were rarely landed in commercial fisheries until the early 1990s with the advent of the live-fish fishery. They are taken in small numbers by commercial hook-and-line and traps for the live –fish market. Kelp rockfish are also taken in sport fisheries, such as spear fishing; their restricted habitat and limited movement make them susceptible to exploitation. Thus, local depressions in the population may occur in areas where diving, skiff fishing, or commercial fishing is concentrated ("Nearshore fishery management plan" 2002).

Blue rockfish are one of the most important recreational species in California for anglers fishing from skiffs and commercial passenger fishing vessels. They are the most frequently caught rockfish north of Point Conception and are the main nearshore recreational fishery in central California. In 2007 a stock assessment for blue rockfish indicated the population is at 29.9% of unfished biomass, which is the precautionary zone for management (Key et al. 2008).

Black rockfish are a major component of recreational catch in Northern California and less common south of Cape Mendocino. An assessment of the black rockfish fishery in 2015 indicates the California stock is at 33% of the unfished biomass which is considered in the precautionary zone for management, however the population has been increasing since the late 1990s (Cope et al. 2015b).

Gopher rockfish were assessed in 2005 and did not appear to be below target levels at the time (Key et al. 2005). The most recent assessment of lingcod indicated the stock has recovered from prior overfishing (Hamel et al. 2009).

Copper rockfish is an important species of the commercial live-fish fishery and recreational catch in skiff and commercial passenger fishing vessels. A 2013 stock assessment for data-moderate stocks indicated that the copper rockfish stock is near target biomass for the stock from Point Conception to Astoria and above target biomass for the stock south of Point Conception. (Cope et al. 2015a).

California scorpionfish biomass declined before 1980, however has since increased to a healthy state and recently catch has been below management limits in most years (Monk et al. 2017).

Brown rockfish are an important component of the nearshore recreational fishery and commercial fishery especially north of Point Conception. The recent stock assessment for brown rockfish indicates that the stock is near target biomass (Cope et al. 2015a).

Yellowtail rockfish biomass estimates were above targeted levels (Cope et al. 2015a). The stock assessment for vermillion rockfish indicates that the stocks experienced overfishing in the early 1990s and depleted abundance into the late 1990s though the stocks are no longer overfished due to management (MacCall 2005).

Bocaccio was formally designated as overfished in 1999 and fishing was severely restricted on this stock and remains restricted (He and Field 2017). The latest stock assessment for cabezon indicates the stock in northern California was calculated to be 45% of its unfished biomass and the southern California sub-stock was calculated to be 60% of its unfished biomass (Cope and Key 2009).

China rockfish are valuable component of the live fish fishery. The assessment of China rockfish indicates that the central stock from the northern California border to Cape Mendocino is at 62% of unfished biomass, which is healthy for management purposes, and the Southern stock from Cape Mendocino to the southern Mexico border was 30% of unfished biomass, which is in the precautionary zone, but has been increasing since 2000 (Cope et al. 2015a).

Kelp greenling is fished for the live fish fishery comprising nearly 45% of the live-fish business in some markets. No estimate of abundance of kelp greenling has been conducted for California, thus parameter estimates from Oregon were used in this study (Berger et al. 2015). Fishing intensity is estimated to have been below the management target level (Berger et al. 2015).

California sheephead was assessed in 2004 and indicated that current unfished mature male and female spawning potential ratio had been reduced to 20% of the unfished condition and a reduction of catch was applied (Alonzo et al. 2004).

The red sea urchin is one of California's most valuable fisheries developing in the 1970s to cater mainly to the Japanese export market. The fishery has been over-exploited in California and considered overfished and was added to the restricted access program in the early 2000s (Kalvass and Roger-Bennett 2001).

Kelp bass are an important recreationally fished species closely associated with kelp habitat.  Kelp bass ranks in the top ten sport fish caught by commercial passenger fishing vehicles in southern California, and increased exploitation rates from 1993-2003 affected mainland kelp bass populations though long-standing regulations appeared to sustain the fishery (Jarvis et al. 2014).

Olive rockfish and black and yellow rockfish have declined in abundance south of Point Conception, though stock assessment has not been completed to assess these species (Wright 2002).

References:

Alonzo, S., M. Key, T. Ish, and A. MacCall. 2004. Status of the California Sheephead (Semicossyphus pulcher) stock. National Marine Fisheries Service Office of Science and Technology.

Berger, A., L. Arnold, and B. Rodomsky. 2015. Status of Kelp Greenling (Hexagrammos decagrammus) along the Oregon Coast in 2015. Pacific Fishery Management Council.

Cope, J., E. Dick, A. MacCall, M. Monk, B. Soper, and C. Wetzel. 2015a. Data-moderate stock assessments for brown, China, copper, sharpchin, stripetail, and yellowtail rockfishes and English and rex soles in 2013. Pacific Fishery Management Council.

Cope, J., D. Sampson, A. Stephens, M. Key, P. Mirick, M. Stachura, T. Tien-shui, P. Weyland, A. Berger, T. Buell, E. Councill, E. Dick, K. Fenske, M. Monk, and B. Rodomsky. 2015b. Assessments of California, Oregon and Washington stocks of black rockfish (Sebastes melanops) in 2015. Northwest Fisheries Science Center.

Hamel, O., S. Sethi, and T. Wadsworth. 2009. Status and Future Prospects for Lingcod in Waters off Washington, Oregon, and California as Assessed in 2009. Northwest Fisheries Science Center.

Jarvis, E., H. Gliniak, and C. Valle. 2014. Effects of fishing and the environment on. the long-term sustainability of the recreational saltwater cass fishery in southern California. California Fish and Game 100:234–259.

Kalvass, P., and L. Roger-Bennett. 2001. California's Living Marine Resources: A Status Report. The California Department of Fish and Game.

Key, M., A. MacCall, T. Bishop, and B. Leos. 2005. STOCK ASSESSMENT of the GOPHER ROCKFISH (Sebastes carnatus). Pacific Fishery Management Council.

Key, M., A. MacCall, J. Field, D. Aseltine-Neilson, and K. Lynn. 2008. The 2007 Assessment of Blue Rockfish (Sebastes Mystinus) in California. California Department of Fish and game.

MacCall, A. 2005. Assessment of Vermilion Rockfish in Southern and Northern California. Pacific Fishery Management Council.

Monk, M. H., X. He, and J. Budrick. 2017. Status of California Scorpionfish (Scorpaena guttata) Off Southern California in 2017. Pacific Fishery Management Council, Portland, OR.

Nearshore fishery management plan. 2002. . California Department of Fish and game.

Wright, N. 2002. Nearshore fishery management plan. California Department of Fish and Game, Marine Region.
