# Supplementary material for: Setting expected timelines of fished population recovery for the adaptive management of a marine protected area network
Source: Ecol Appl. 2019 Jul 26;29(6):e01949. doi: 10.1002/eap.1949 (PMC9285580; doi:10.1002/eap.1949)
Supplement: Supplementary file 2 [file EAP-29-e01949-s003.pdf]

Supporting Information. Katherine A. Kaplan, Lauren Yamane, Louis W. Botsford, Marissa L. Baskett, Alan Hastings, Sara Worden, J. Wilson White. 2019. Setting expected timelines of fished population recovery for the adaptive management of a marine protected area network. *Ecological Applications.*

Appendix S2. Expected transient increase in biomass, as compared to abundance, with implementation of MPAs

There is an expectation that biomass will increase by a greater amount than abundance immediately following the implementation of an MPA, primarily because it includes the increase in size of individual fish, in addition to the increase in abundance. Here we examine this question by calculating the change in both abundance and biomass from an age-structured model of fished population. This analysis covers the immediate population response of the "filling in" of the age structure, not any potential longer-term effects on recruitment.

The change in abundance has been presented earlier (White, et al. 2013) in terms of the ultimate ratio of abundance to initial abundance,

$$\frac{N(t)}{N(0)} = \frac{M+F}{M} \qquad \text{(S1)}$$

Where N(t) is the abundance of individuals older than the age of first capture a_c, t time units after implementation, M is the natural mortality rate, and F is the fishing mortality rate. This result is taken from,

$$\frac{N(t)}{N(0)} = (M+F)\left[\frac{1}{M} - \left(\frac{1}{M} - \frac{1}{M+F}\right)e^{-Mt}\right] = \frac{M+F}{M} - \left[\left(\frac{1}{M} - \frac{1}{M+F}\right)e^{-Mt}\right] \qquad \text{(S2)}$$

in Appendix 2 of that publication. This expression has a simple intuitive interpretation. The abundance per recruit for a fished population is 1/(M+F), whereas the abundance per

recruit for an unfished population is 1/M. In the long term, following protection by an MPA, one would expect the abundance to increase by that ratio of those two quantities (i.e., (M+F)/M). The RHS of equation (S2) is that quantity minus the difference between those two quantities decaying exponentially at a rate M. Thus, the ratio of abundances would increase as an inverted exponential decay.

The derivation of equation (S2) was based on the observation that following the implementation of an MPA, changing F from the fished value to zero will separate the age structure into two parts: those recruited to the fished population after the implementation of the MPA, and those recruited prior to the implementation. These will be separated at a_c+t, where a_c is the age of first capture. We can determine the abundance of the younger ages from,

$$N_{low}(t) = \int_{a_c}^{a_c+t} e^{-Ma}\,da = \frac{1}{M}\left(e^{-Ma_c} - e^{-M(a_c+t)}\right) \qquad \text{(S3a)}$$

and of the older ages from,

$$N_{high}(t) = \int_{a_c+t}^{\infty} e^{-Ma} e^{-(M+F)\left(a-(a_c+t)\right)}\,da = \frac{e^{-M(a_c+t)}}{M+F} \qquad \text{(S3b)}$$

We will normalize these by dividing by the initial abundance, just before implementation,

$$N(0) = N_{low}(0) + N_{high}(0) = \frac{e^{-M(a_c)}}{M+F}$$

The normalized total abundance can then be written as

$$\frac{N_{low}(t)+N_{high}(t)}{N(0)} = \frac{M+F}{M}\left(1 - e^{-Mt}\right) + e^{-Mt} =$$

$$(M+F)\left[\frac{1}{M} - \left(\frac{1}{M} - \frac{1}{M+F}\right)e^{-Mt}\right] \qquad \text{(S4)}$$

We can write expressions for the increase in biomass that are similar to these for the increase in abundance by representing growth in terms of length in the von Bertalanffy form and weight as length cubed. For each age a biomass will be

$$B(a) = L_\infty{}^3[1 - e^{-kt}]^3 = L_\infty{}^3 \sum_{i=0}^{3} K_i e^{-ika} \qquad \text{(S5)}$$

where K_i=1, -3, 3, and -1 for i=0, 1, 2, and 3. Inserting this into the integrand of equation (S3a), the expression for biomass, B_{low} would be

$$B_{low}(t) = L_\infty{}^3 \sum_{i=0}^{3} K_i \int_{a_c}^{a_c+t} e^{-Ma}\, e^{-ika}\; da = L_\infty{}^3 \sum_{i=0}^{3} K_i \frac{e^{-(M+ik)a_c}\left(1-e^{-(M+ik)t}\right)}{M+ik} \qquad \text{(S6a)}$$

and the expression for $B_{high}(t)$ would be

$$B_{high}(t) = L_\infty{}^3 \sum_{i=0}^{3} K_i \int_{a_c+t}^{\infty} e^{-Ma} e^{-(M+F)(a-(a_c+t))}\, e^{-ika}\; da = L_\infty{}^3 \sum_{i=0}^{3} K_i \frac{e^{-(M+ik)(a_c+t)}}{M+F+ik}$$

(S6b)

Similarly, the expression for B(0) would be the same as S6b, with *t* = 0.

$$B(0) = L_\infty{}^3 \sum_{i=0}^{3} K_i \int_{a_c}^{\infty} e^{-Ma} e^{-(M+F)(a-a_c)}\, e^{-ika}\; da = L_\infty{}^3 \sum_{i=0}^{3} K_i \frac{e^{-(M+ik)a_c}}{M+F+ik}$$

(S7)

Thus,

$$\frac{B_{low}(t)+B_{high}(t)}{B(0)} = \frac{\sum_{i=0}^{3} K_i \frac{e^{-(M+ik)a_c}\left(1-e^{-(M+ik)t}\right)}{M+ik} + \sum_{i=0}^{3} K_i \frac{e^{-(M+ik)(a_c+t)}}{M+F+ik}}{\sum_{i=0}^{3} K_i \frac{e^{-(M+ik)a_c}}{M+F+ik}} \qquad \text{(S8)}$$

Then, in the limit as *t* becomes very large, the expression becomes

$$\frac{B_{low}(t)+B_{high}(t)}{B(0)} = \frac{\sum_{i=0}^{3} K_i \frac{e^{-(M+ik)a_c}}{M+ik}}{\sum_{i=0}^{3} K_i \frac{e^{-(M+ik)a_c}}{M+F+ik}} \qquad \text{(S9)}$$

This expression for the increase in biomass can be compared to the similar version in equation (S4). As in equation (S4), this version has a term similar to $1/M$ in the numerator and one similar to $1/(M+F)$ in the denominator, so one can see a parallel to the $(M+F)/M$ solution, even if the whole expression is a bit more complicated.

To gain a basic understanding of how Eq. (S9) differs from Eq. (S4), note that the coefficients, *i*, in the cubic binomial expansion appear in a negative exponent, so they will gradually decline in magnitude (and in their contribution to the solution). In the first

step in the sequence, $i = 0$, has $1/M$ in the numerator and $1/(M+F)$ in the denominator, so in isolation it would produce the original $(M+F)/M$ result. The second step in the sequence, $i = 1$ will have the next greatest influence on the magnitude of the result. It is associated with the coefficient $K_i = -3$, so it has an effect similar to adding a negative number to the numerator and denominator of the $(M+F)/M$ ratio. Adding the same negative number to both sides of a ratio will increase a ratio, which explains in part why the biomass ratio will always be greater than the abundance ratio. Additionally, the time scale for the rate of increase for abundance (i.e., $1/M$) is increased by the addition of the ik terms, thus making the time scale of the biomass response faster than that of abundance following implementation of an MPA.
