# Supplementary material for: Setting expected timelines of fished population recovery for the adaptive management of a marine protected area network
Source: Ecol Appl. 2019 Jul 26;29(6):e01949. doi: 10.1002/eap.1949 (PMC9285580; doi:10.1002/eap.1949)

Supporting Information. Katherine A. Kaplan, Lauren Yamane, Louis W. Botsford, Marissa L. Baskett, Alan Hastings, Sara Worden, J. Wilson White. 2019. Setting expected timelines of fished population recovery for the adaptive management of a marine protected area network. *Ecological Applications.*

Appendix S3. Supplementary tables and figures.

Table S1. Length of time in years to reach 95% of final equilibrium abundance (*N* ratio) or biomass ratio (*B* ratio) for deterministic open population models.

Species	*N* ratio time to equilibrium (y)	*B* ratio time to equilibrium (y)	Final *N* ratio (N_t/N_0)	Final *B* ratio (B_t/B_0)
Kelp rockfish	10	13	1.69	2.24
Blue rockfish	17	21	2.04	2.94
Black rockfish	12	14	1.32	1.45
Gopher rockfish	10	12	1.69	1.95
Lingcod	9	12	1.72	2.31
Copper rockfish	22	27	1.79	2.38
CA scorpionfish	8	10	1.57	1.92
Brown rockfish	15	18	1.76	2.47
Yellowtail rockfish	18	22	1.52	1.81
Vermillion rockfish	24	31	2.23	3.94
Bocaccio	1	4	1.06	1.08
Cabezon	6	7	1.33	1.45
China rockfish	40	41	2.45	2.62
Kelp greenling	6	8	1.44	1.60
CA sheephead	9	12	1.78	2.22
Red sea urchin	35	37	4.63	6.71
Kelp bass	11	16	1.55	2.12
Olive rockfish	12	14	1.42	1.64
Black & yellow rockfish	9	9	1.65	1.68

Table S2. Estimates of transient metrics given closed population dynamics; period of oscillations (P), rate of convergence to asymptotic behavior (ρ), the similarity to stable age distribution (θ) and the time to convergence (1/log(ρ)).

Species	P (years)	ρ	θ (°)	1/log(ρ) (years)
Kelp rockfish	5.94	1.29	6.59	11.69
Blue rockfish	10.20	1.19	9.21	17.45
Black rockfish	11.54	1.15	2.79	21.1
Gopher rockfish	7.10	1.31	5.96	11.1
Lingcod	5.88	1.36	7.42	9.81
Copper rockfish	22.02	1.12	5.99	25.94
California scorpionfish	6.51	1.35	5.31	9.95
Brown rockfish	14.89	1.19	6.72	17.3
Yellowtail rockfish	12.15	1.14	3.84	23.1
Vermillion rockfish	12.36	1.13	7.28	24
Bocaccio	46.94	1.17	0.44	19.34
Cabezon	5.30	1.32	3.30	10.69
China rockfish	71.75	1.11	11.19	29.09
Kelp greenling	4.88	1.39	4.16	9.13
California sheephead	7.11	1.31	6.65	11.16
Red sea urchin	8.17	1.29	27.17	13.04
Kelp bass	14.51	1.20	5.82	16.47
Olive rockfish	24.23	1.18	3.43	17.75
Black & yellow rockfish	7.40	1.25	1.56	13.19

Figure S1. Age distribution of nearshore fished species before and after MPA implementation. Red shading indicates the fished population at equilibrium; the remaining colors denote a previously fished population in the first 5-50 years of protection inside an MPA. Simulations assume open population dynamics with deterministic recruitment.

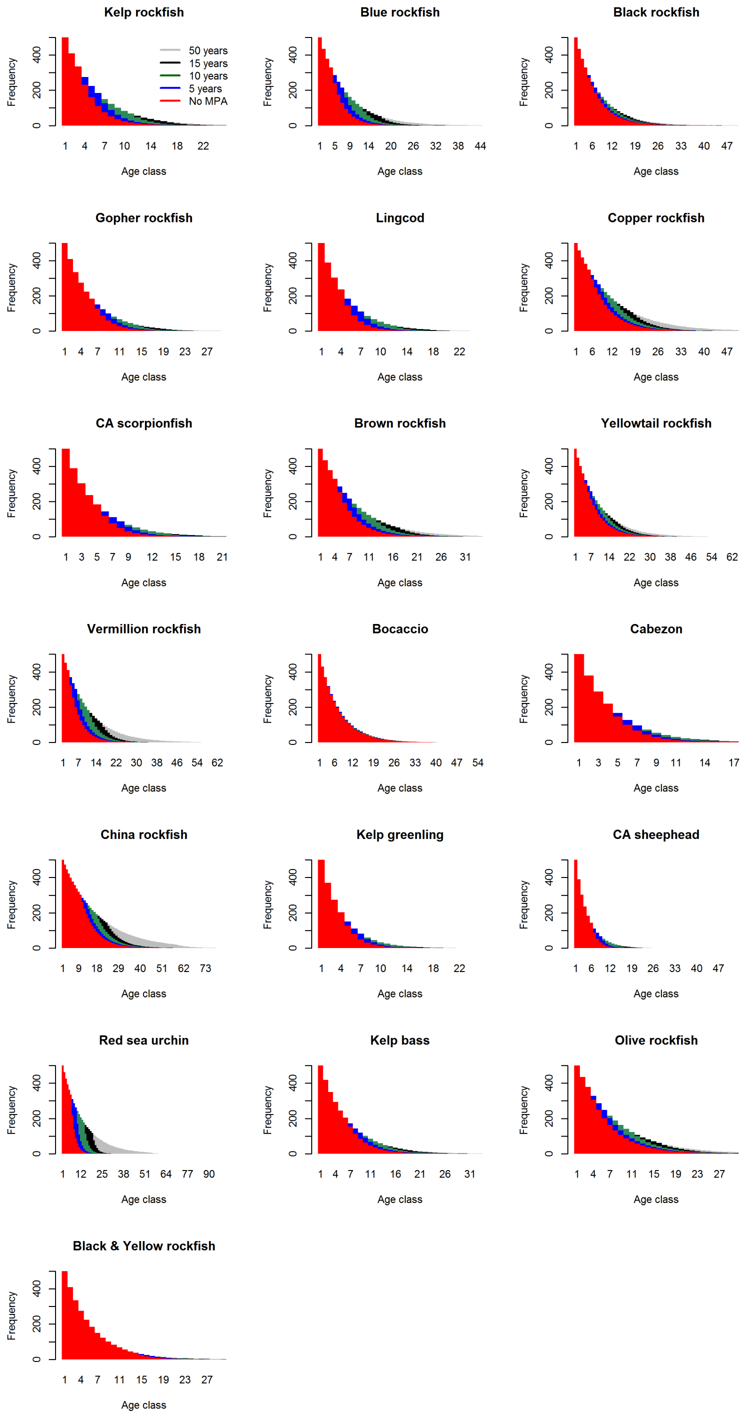

Figure S2. Abundance (red) and biomass (blue) ratio increases over time modeled as a deterministic open population with constant recruitment for 19 species modeled in this study.

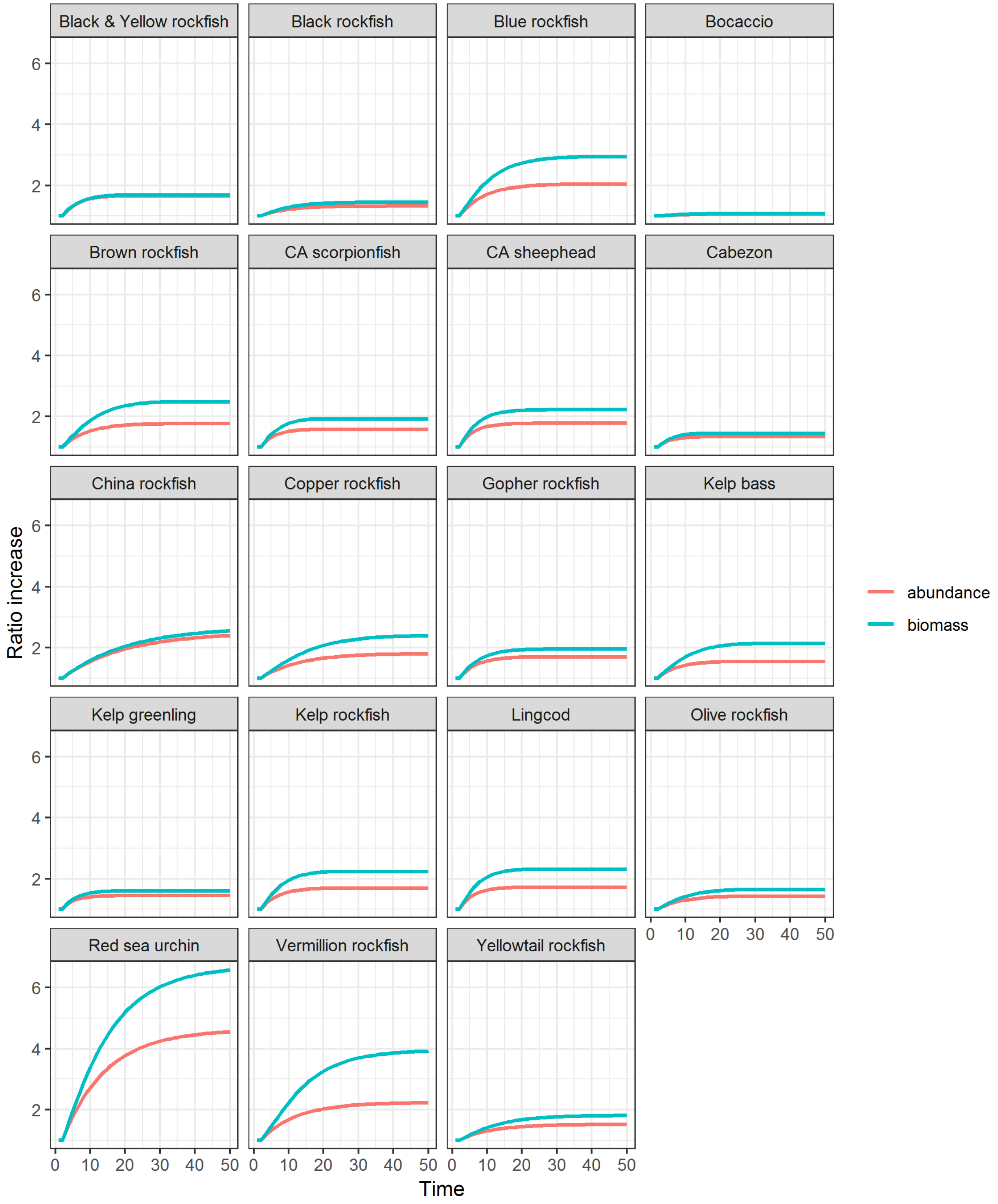

Figure S3. Trajectories of population abundance ratios over time, with (blue) and without (red) MPA establishment, given open population dynamics with stochastic recruitment. Banded intervals represent the lower quartile and upper quartile for 500 simulated runs, and solid colored lines indicate median responses. Greater separation between the ranges of outcomes with versus without MPA establishment indicates greater ability to distinguish an MPA effect. Black dotted lines represent deterministic model projections inside an MPA.

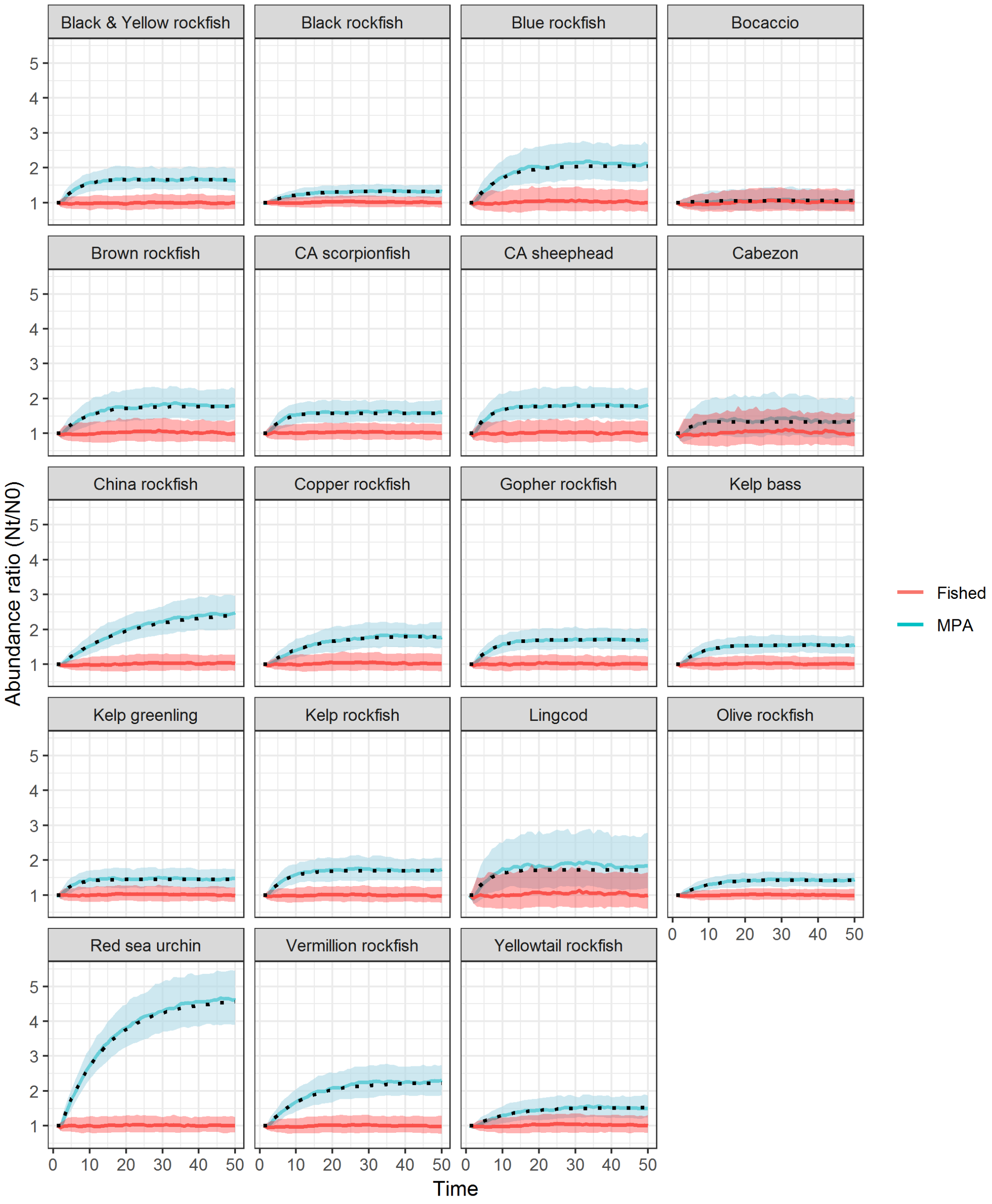

Figure S4. Trajectories of population biomass ratios over time, with (blue) and without (red) MPA establishment, given open population dynamics with stochastic recruitment. Banded intervals represent the lower quartile and upper quartile for 500 simulated runs, and solid colored lines indicate median responses. Greater separation between the ranges of outcomes with versus without MPA establishment indicates greater ability to distinguish an MPA effect. Black dotted lines represent deterministic model projections inside an MPA.

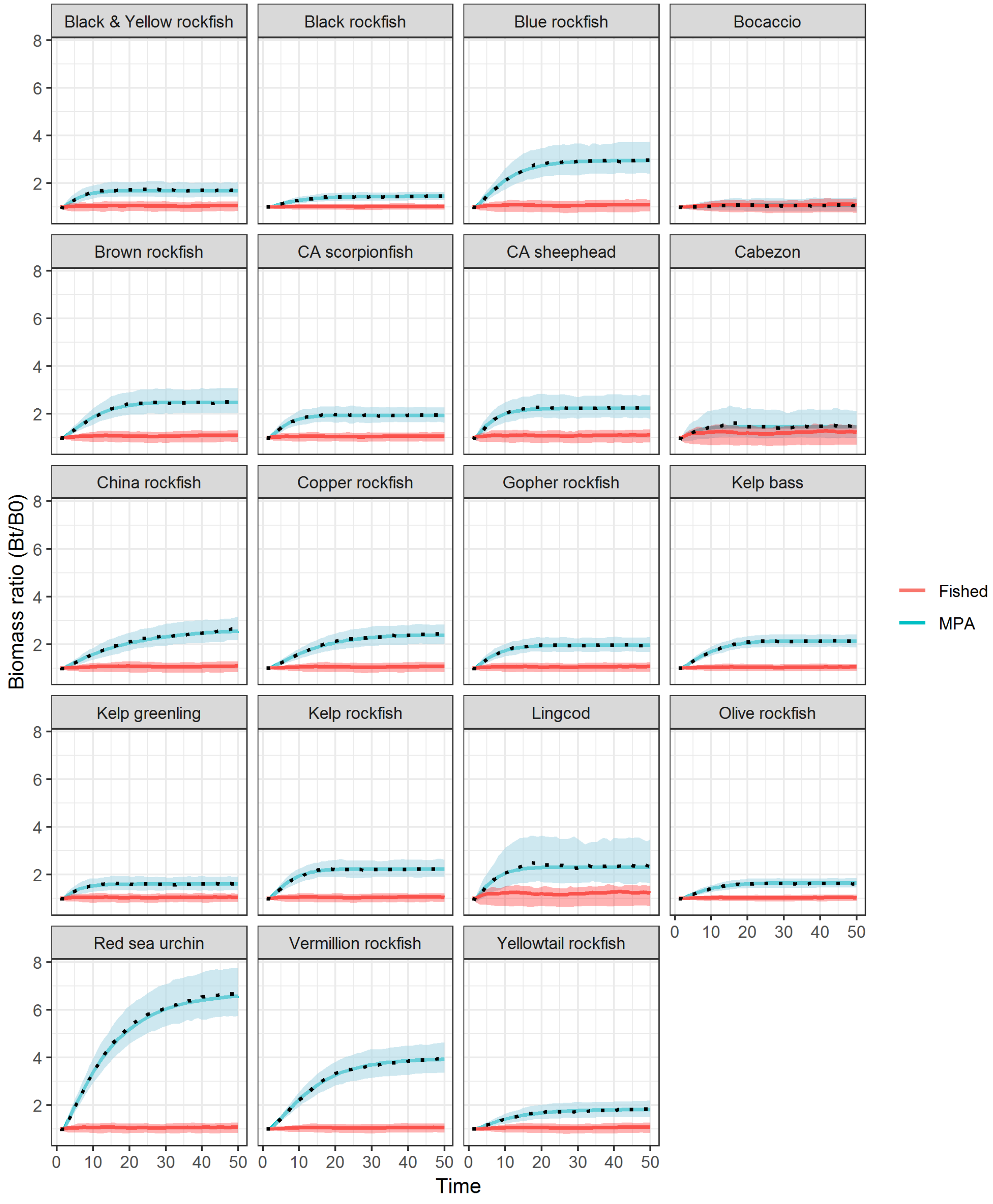

Figure S5. MPA response detectability, measured as area under the ROC curve (AUC) for different species at different time points since MPA implementation for (a) changes in abundance and (b) biomass in the MPA as compared to the fished state. Simulations assume open population dynamics with stochastic recruitment.

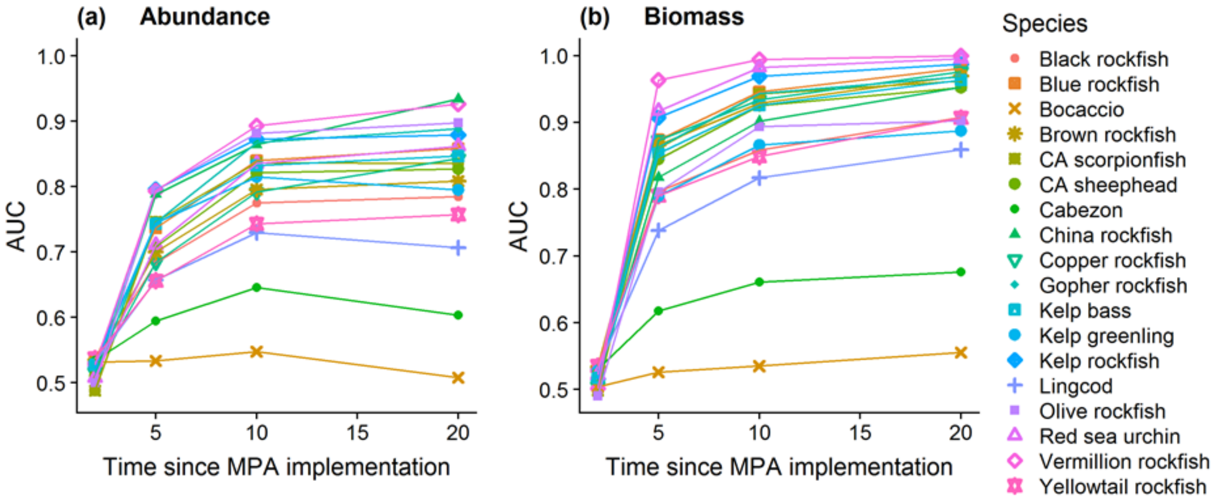

Figure S6. Abundance ratio changes receiver-operating characteristic (ROC) curves in an MPA compared to fished state in 2 (black), 5 (red), 10 (green) and 20 (blue) years after MPA implementation for nearshore species. Simulations assume open population dynamics with stochastic recruitment.

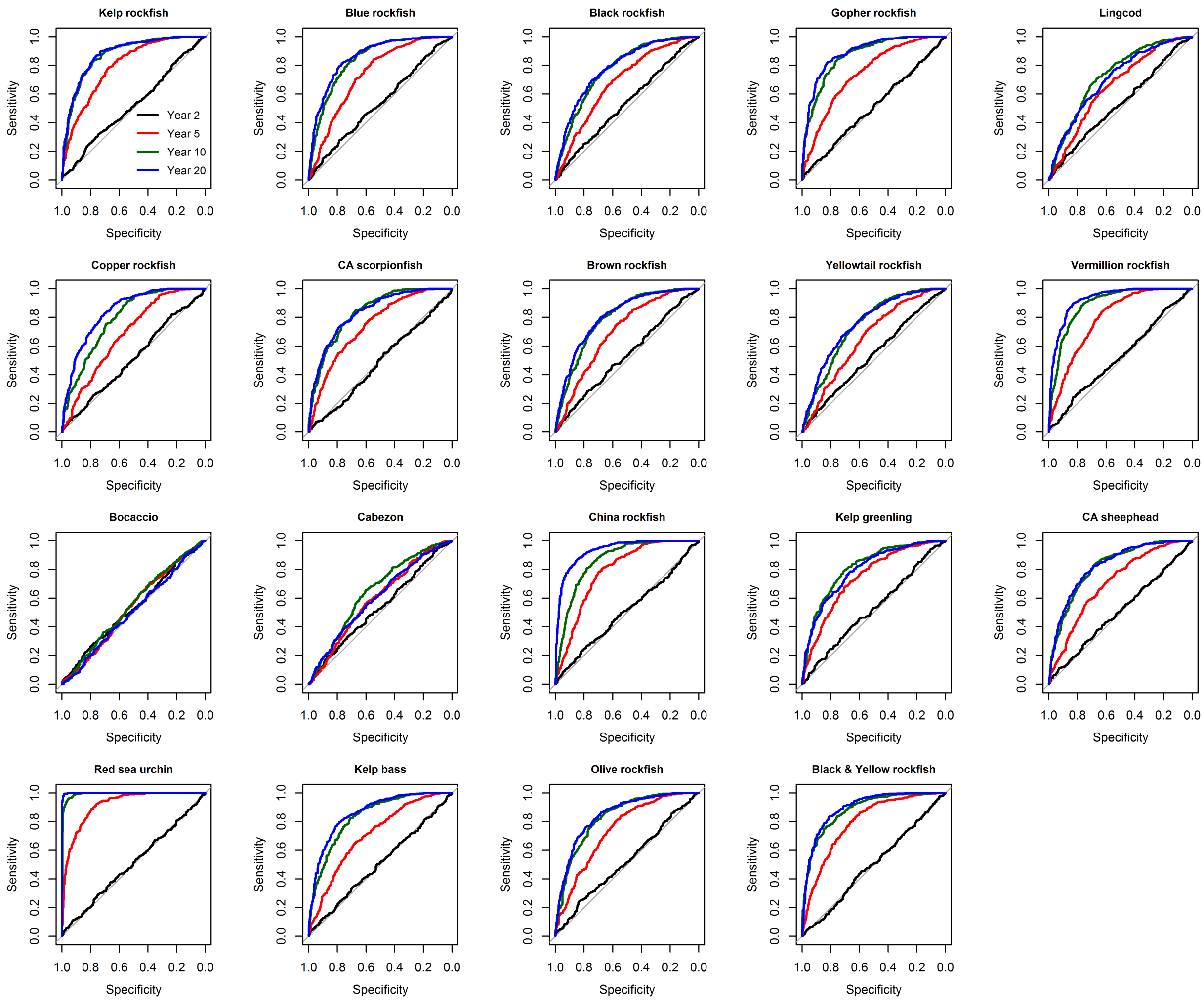

Figure S7. Biomass ratio changes receiver-operating characteristic curves in an MPA compared to fished state in 2 (black), 5 (red), 10 (green) and 20 (blue) years after MPA implementation for nearshore species. Simulations assume open population dynamics with stochastic recruitment.

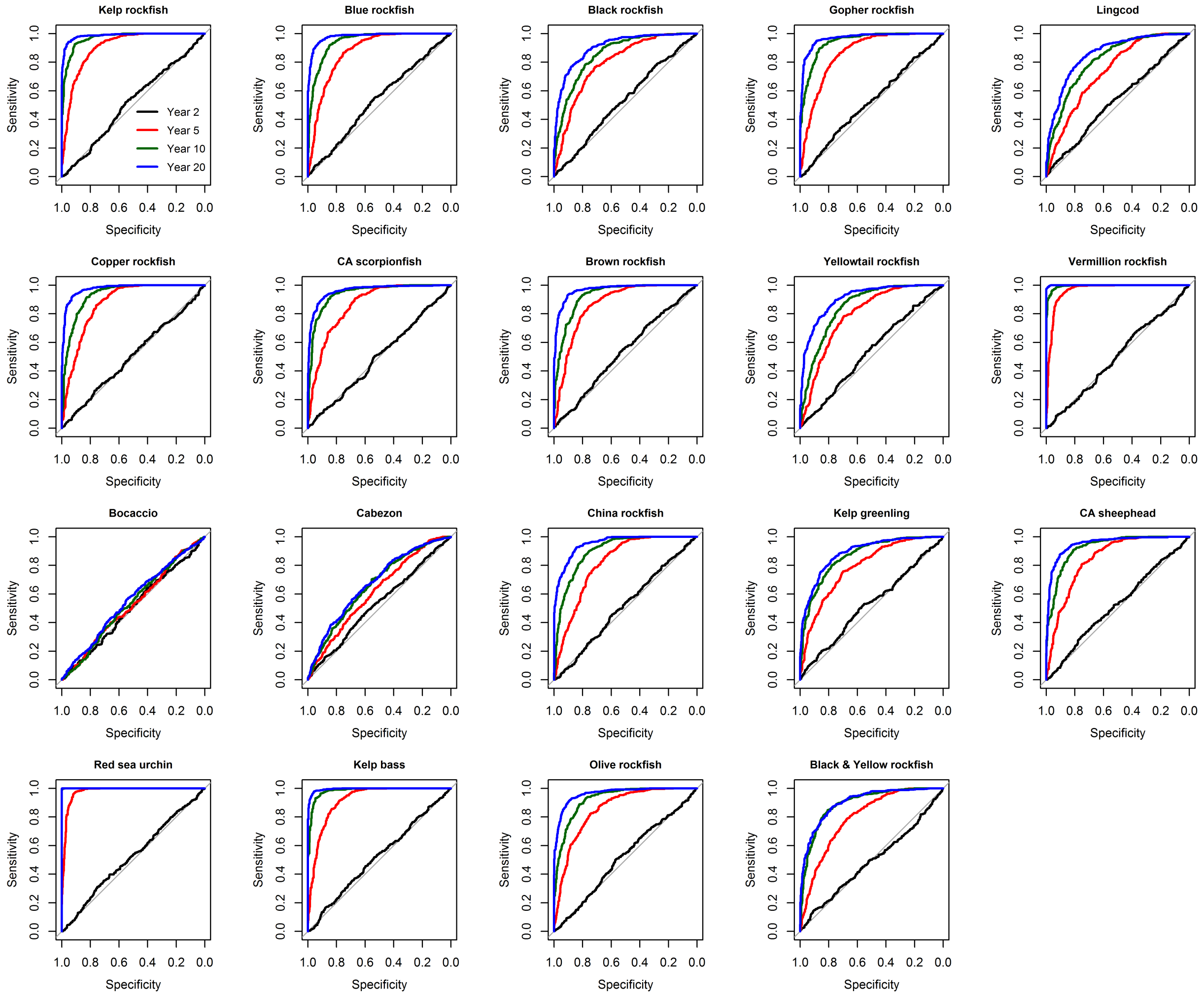

Figure S8. Abundance (red) and biomass (blue) ratio changes in response to MPA implementation for closed populations with deterministic recruitment.

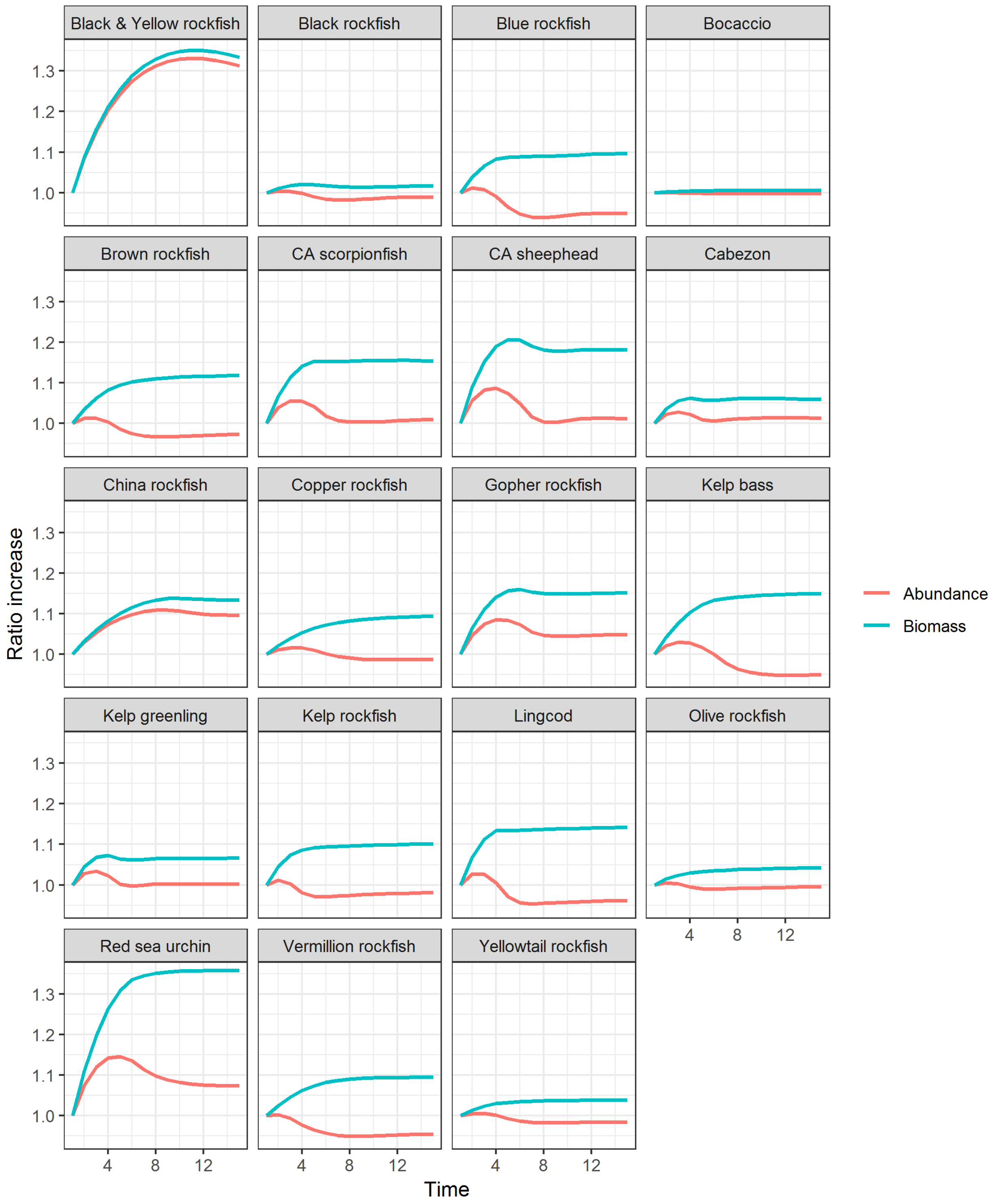

Supplement: Supplementary file 3 [file EAP-29-e01949-s002.pdf]
