# Supplementary material for: Setting expected timelines of fished population recovery for the adaptive management of a marine protected area network
Source: Ecol Appl. 2019 Jul 26;29(6):e01949. doi: 10.1002/eap.1949 (PMC9285580; doi:10.1002/eap.1949)
Supplement: Supplementary file 4 [file EAP-29-e01949-s001.pdf]

Supporting Information. Katherine A. Kaplan, Lauren Yamane, Louis W. Botsford, Marissa L. Baskett, Alan Hastings, Sara Worden, J. Wilson White. 2019. Setting expected timelines of fished population recovery for the adaptive management of a marine protected area network. *Ecological Applications.*

Appendix S4. Transient response of a fished population to an MPA: the role of recruitment variability

The theory describing short-term ("transient") MPA responses by White et al. (2013) was premised on larval recruitment to the open population being constant over time. If we relax that assumption, we must describe both the average expectation for increase over time after the MPA as well as the variance around that expectation. Assume that annual recruitment follows a log-normal distribution obtained by exponentiating a normal distribution with mean □$_R$ and standard deviation □$_R$. The standard deviation of the resulting distribution is

$$\sigma = \left[\left(e^{\sigma_R^2} - 1\right)e^{\left(2\mu_R + \sigma_R^2\right)}\right]^{0.5} \qquad \text{(S1)}$$

As before, we will express abundance relative to the pre-MPA abundance at time $t = 0$, N_0 (calculated as the summed abundance across all age classes). Thus each year the standard deviation around the expected relative abundance will increase as another cohort of recruits (drawn from the lognormal population) enters the population. If the standard deviation of the recruits (age class 0) is □ then the standard deviation of one-year olds will be □e^{-M}, because both the mean and the standard deviation of the recruit cohort will be reduced by natural mortality.

Thus the variance of subsequent age class *a* will be □e^{-Ma}. Once the transient is complete and the age structure is completely filled in, the standard deviation of the summed abundance of all age classes (*A*) can be obtained by summing the variance in each age class:

$$\sigma_{total} = \left[\sum_{a=0}^{A}(\sigma e^{-Ma})^2 + \sum_{i \neq j}^{A} \sigma_{ij}\right]^{0.5} \qquad \text{(S2)}$$

where □$_{ij}$ is the covariance between recruitment into the ith and j^{th} age classes. If there is no interannual correlation in recruitment, that term can be ignored (but it becomes important if there is, for example, environmentally-driven temporal autocorrelation in recruit year-class strength). During the transient, the variance of the summed abundance will gradually increase as new recruits arrive and the age structure fills in. At any time after MPA implementation, the standard deviation will be

$$\sigma_{total}(t) = [\sum_{a=0}^{t}(\sigma e^{-Ma})^2]^{0.5} \qquad \text{(S3)}$$

and the standard deviation of just the fished age classes is

$$\sigma_{total}(t) = \left[\sum_{a=0}^{t}\left(\sigma e^{-M(a+a_c)}\right)^2\right]^{0.5} \qquad \text{(S4)}$$

where a_c is the age of entry to the fishery.

From these equations we can see that the two factors affecting variation around the mean response are the standard deviation of recruitment, □, and the natural mortality rate, *M*, which

determines the rate at which recruitment variation is attenuated in subsequent age classes (Fig. S1). A numerical simulation (200 replicates) for two different pairs of *M* and □ values, using life history parameters for kelp rockfish (Table 1), shows a good match to the analytical prediction (Eq. S4; Fig. S2).

References

White JW, Botsford LW, Hastings A, Baskett ML, Kaplan DM, Barnett LAK. 2013. Transient responses of fished populations to marine reserve establishment. Conservation Letters 6:180-191

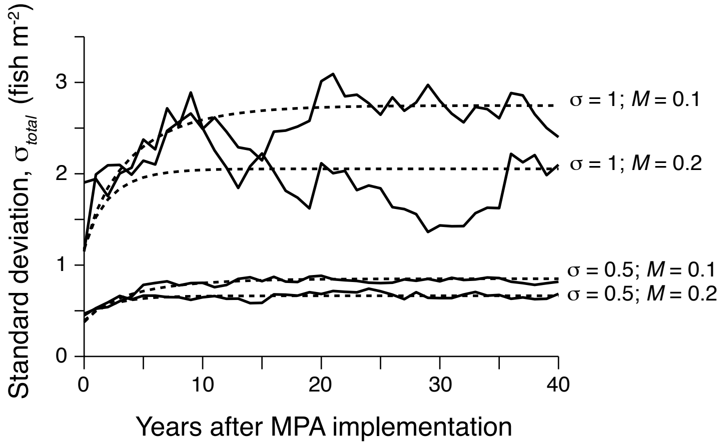

Fig. S1. Analytical prediction (dashed curves) and numerical simulation (200 replicates; solid curves) for the standard deviation of the relative abundance of fished age classes after MPA implementation (relative to abundance at t = 0). Results are shown for indicated values of the standard deviation of recruitment, □$_R$, and natural mortality rate, *M*.

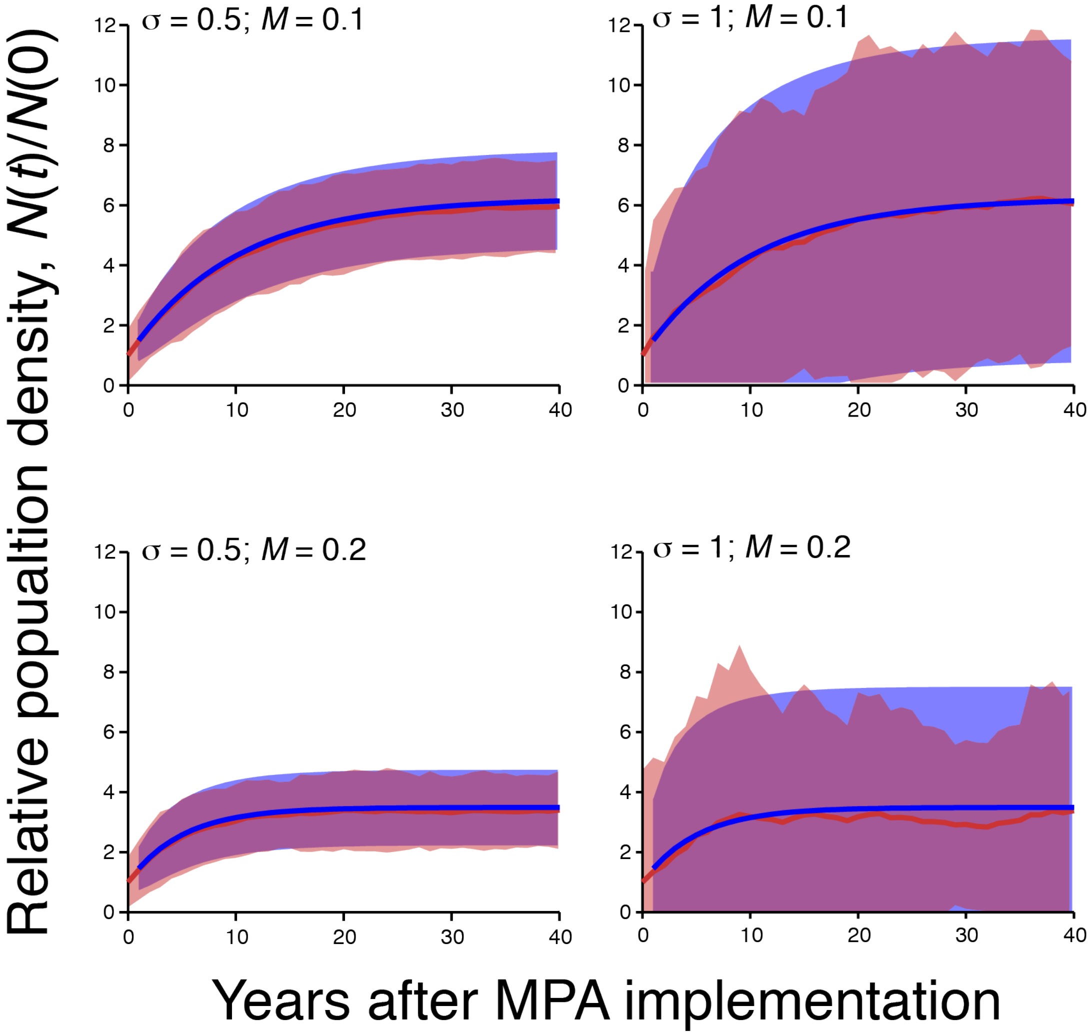

Fig. S2. Analytical prediction (blue) and numerical simulation (200 replicates; red) for the mean (solid curves) and confidence region (calculated as 1.96*standard deviation; shaded regions) for the relative abundance of fished age classes after MPA implementation (relative to abundance at $t = 0$). Results are shown for indicated values of the standard deviation of recruitment, □, and natural mortality rate, *M*.
