# Supplementary material for: Setting expected timelines of fished population recovery for the adaptive management of a marine protected area network
Source: Ecol Appl. 2019 Jul 26;29(6):e01949. doi: 10.1002/eap.1949 (PMC9285580; doi:10.1002/eap.1949)

Katherine A. Kaplan; Lauren Yamane; Louis W. Botsford; Marissa L. Baskett; Alan Hastings; Sara Worden, J. Wilson White. Setting expected timelines of fished population recovery for the adaptive management of a marine protected area network. *Ecological Applications*

Appendix S5. Stochastic recruitment for closed population dynamics

We modeled a closed population with stochastic recruitment as represented by variability in the larval survival parameter (α) of the closed model. To model stochasticity, we assume larval survival follows a log-normal distribution with a mean of α and standard deviation (σ) (Table 1). We modeled the stochastic closed population runs such that the median of 500 simulations experience no growth in the unfished state (λ=1.0). We show the ratio changes for abundance (Figure S1) and biomass (Figure S2). Adding stochasticity to recruitment in the closed population model has the potential to result in statistical detectability decreasing over time because we are applying a linear population model with variance in the N_t/N_0 or B_t/B_0 ratios increasing with time (Figures S3 and S4). Generally, for a closed population model with stochasticity, detectability of responses does not increase with time as in the open population model.

Figure S1. Median abundance ratio change over time modeled as a closed population with stochastic larval survival. Banded intervals represent the lower quartile and upper quartile for 500 simulated runs.

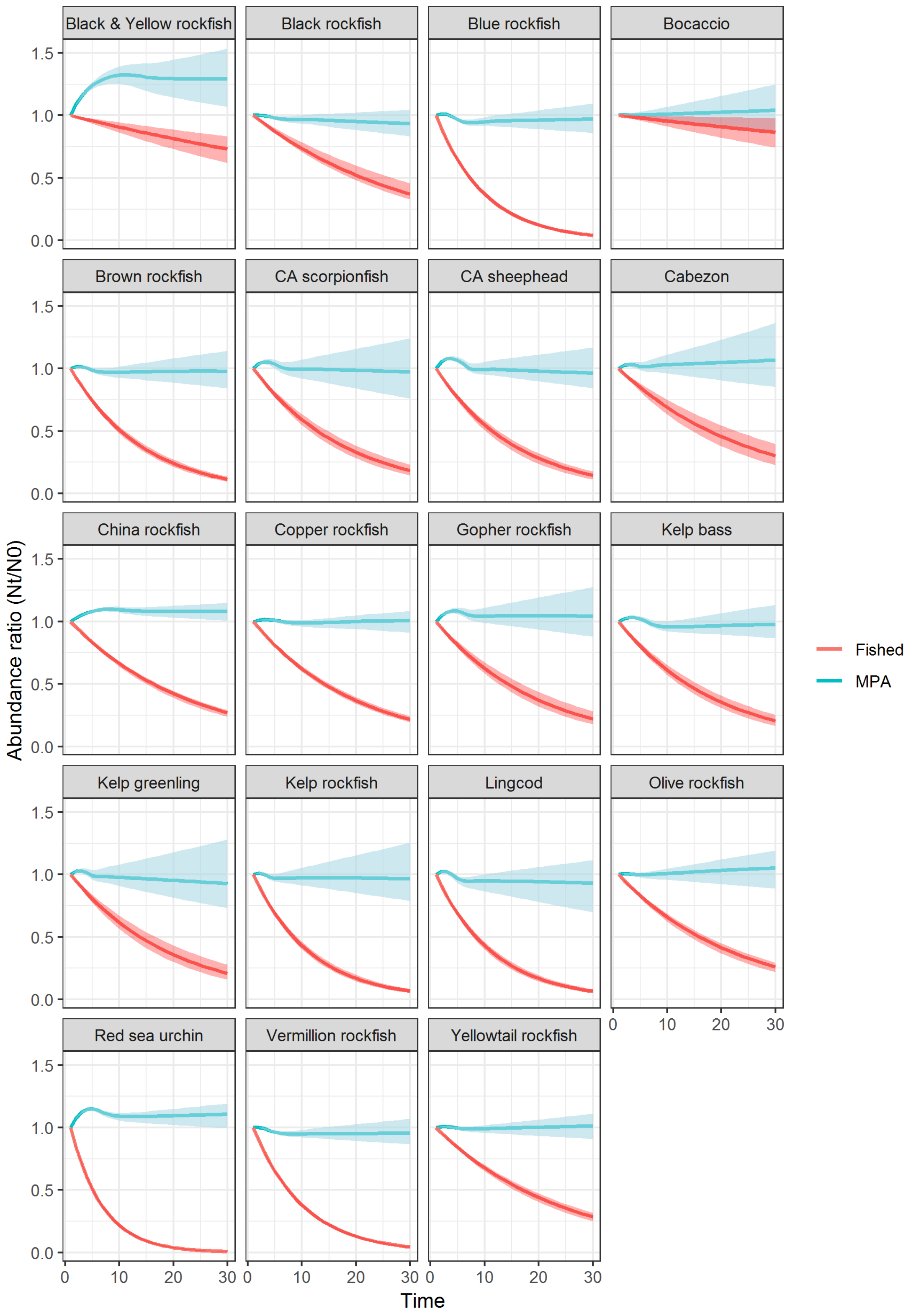

Black & Yellow rockfish
Black rockfish
Blue rockfish
Bocaccio
Brown rockfish
CA scorpionfish
CA sheephead
Cabezon
China rockfish
Copper rockfish
Gopher rockfish
Kelp bass
Kelp greenling
Kelp rockfish
Lingcod
Olive rockfish
Red sea urchin
Vermillion rockfish
Yellowtail rockfish
Abundance ratio (Nt/N0)
Time
Fished
MPA

Figure S2. Median biomass ratio change over time modeled as a closed population with stochastic larval survival. Banded intervals represent the lower quartile and upper quartile for 500 simulated runs.

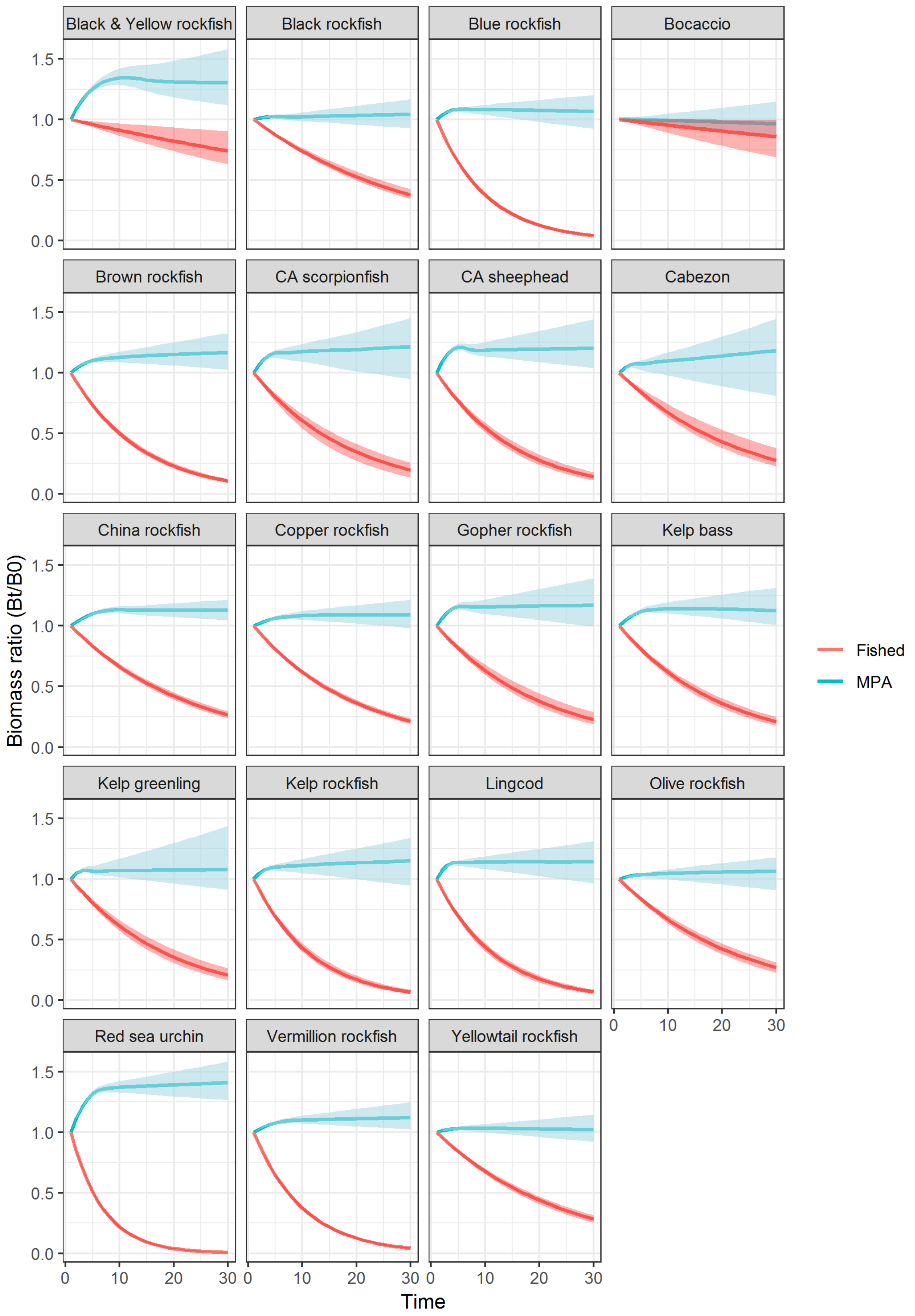

Black & Yellow rockfish
Black rockfish
Blue rockfish
Bocaccio
Brown rockfish
CA scorpionfish
CA sheephead
Cabezon
China rockfish
Copper rockfish
Gopher rockfish
Kelp bass
Kelp greenling
Kelp rockfish
Lingcod
Olive rockfish
Red sea urchin
Vermillion rockfish
Yellowtail rockfish
Biomass ratio (Bt/B0)
0.0
0.5
1.0
1.5
Time
0
10
20
30
Fished
MPA

Figure S3. Receiver-operating characteristic (ROC) curves for abundance ratio changes modeled as a closed population with stochastic larval survival in an MPA compared to fished state for 2 years (black), 5 years (red), 10 years (green) and 20 years (blue) after MPA implementation for nearshore rockfish species.

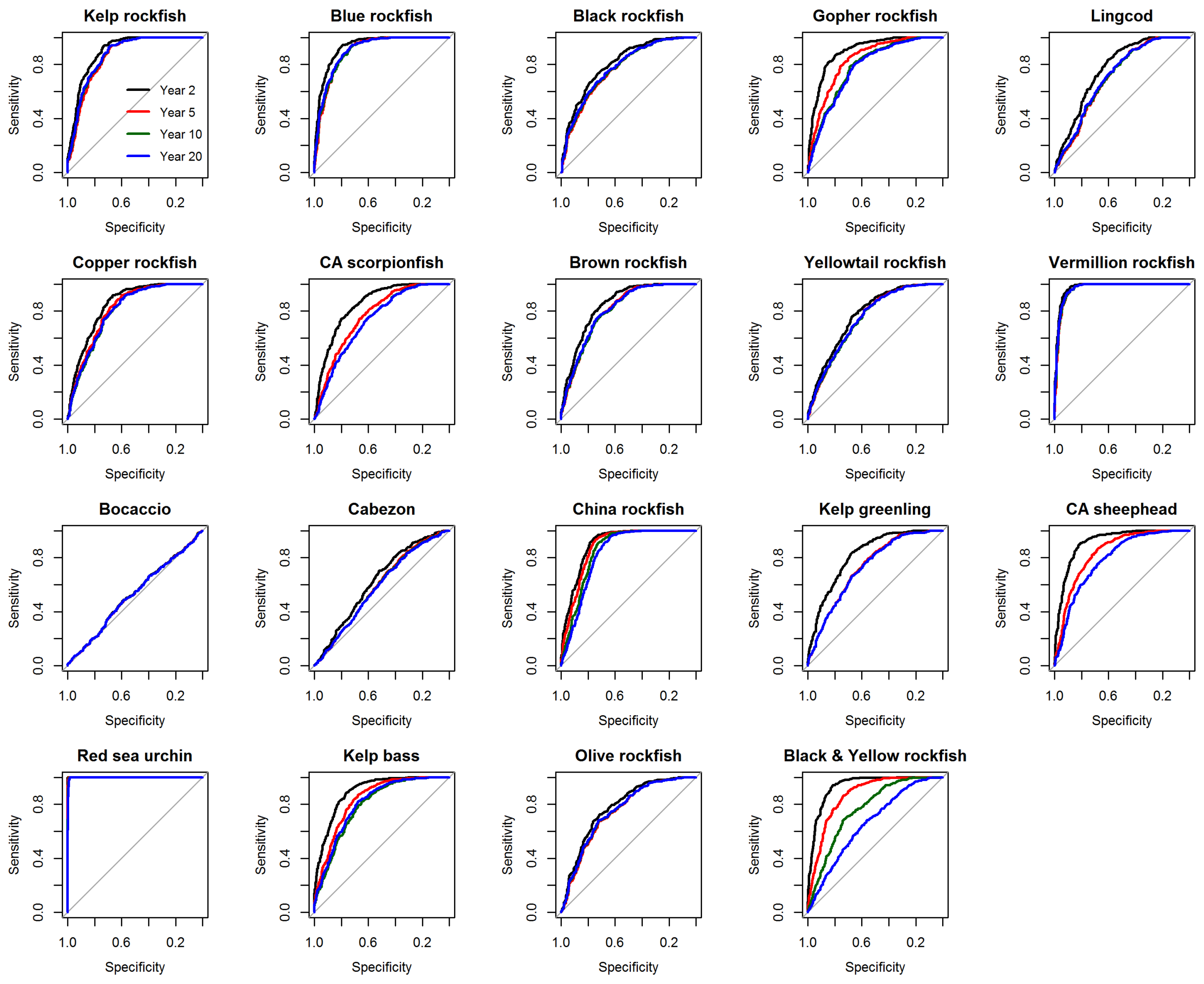

Figure S4. Receiver-operating characteristic (ROC) curves for biomass ratio changes modeled as a closed population with stochastic larval survival in an MPA compared to fished state for 2 years (black), 5 years (red), 10 years (green) and 20 years (blue) after MPA implementation for nearshore rockfish species.

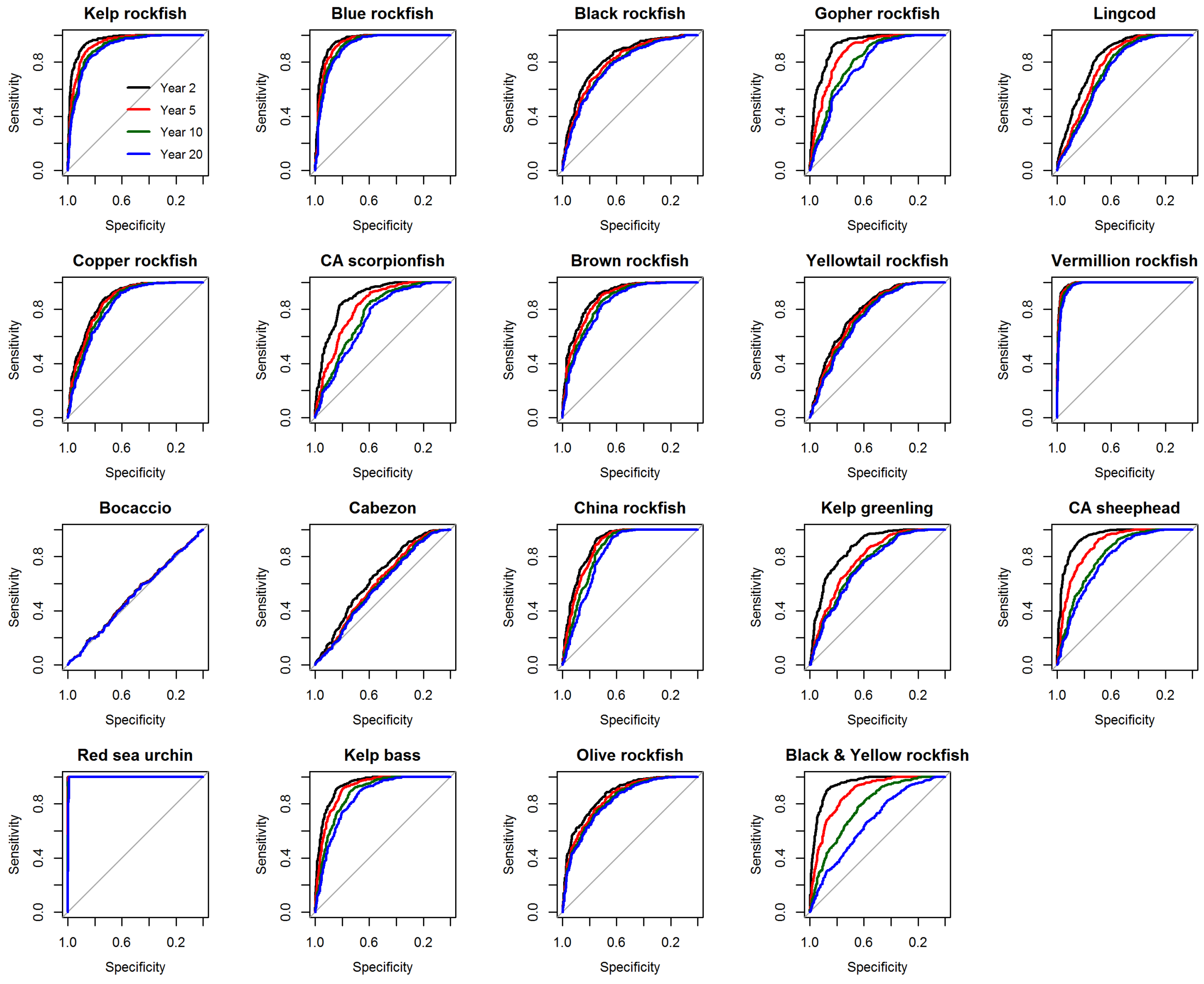

Supplement: Supplementary file 5 [file EAP-29-e01949-s004.pdf]
